# Development of Cell‐Permeable, Non‐Helical Constrained Peptides to Target a Key Protein–Protein Interaction in Ovarian Cancer

**DOI:** 10.1002/anie.201609427

**Published:** 2016-12-05

**Authors:** Mareike M. Wiedmann, Yaw Sing Tan, Yuteng Wu, Shintaro Aibara, Wenshu Xu, Hannah F. Sore, Chandra S. Verma, Laura Itzhaki, Murray Stewart, James D. Brenton, David R. Spring

**Affiliations:** ^1^ Department of ChemistryUniversity of CambridgeLensfield RoadCambridgeCB2 1EWUK; ^2^ Cancer Research UK Cambridge InstituteUniversity of CambridgeLi Ka Shing Centre, Robinson WayCambridgeCB2 0REUK; ^3^ Bioinformatics Institute, Agency for Science, Technology and ResearchA*STAR30 Biopolis Street, #07-01 MatrixSingapore138671Singapore; ^4^ SciLifeLabTomtebodavägen 23A171 65 SolnaStockholmSweden; ^5^ MRC Laboratory of Molecular BiologyFrancis Crick Avenue, Cambridge Biomedical CampusCambridgeCB2 0QHUK; ^6^ Department of PharmacologyTennis Court RoadCambridgeCB2 1PDUK; ^7^ School of Biological SciencesNanyang Technological University60 Nanyang DriveSingapore637551Singapore; ^8^ Department of Biological SciencesNational University of Singapore14 Science Drive 4Singapore117543Singapore

**Keywords:** constrained peptides, drug discovery, nuclear import, peptide therapeutics, peptidomimetics

## Abstract

There is a lack of current treatment options for ovarian clear cell carcinoma (CCC) and the cancer is often resistant to platinum‐based chemotherapy. Hence there is an urgent need for novel therapeutics. The transcription factor hepatocyte nuclear factor 1β (HNF1β) is ubiquitously overexpressed in CCC and is seen as an attractive therapeutic target. This was validated through shRNA‐mediated knockdown of the target protein, HNF1β, in five high‐ and low‐HNF1β‐expressing CCC lines. To inhibit the protein function, cell‐permeable, non‐helical constrained proteomimetics to target the HNF1β–importin α protein–protein interaction were designed, guided by X‐ray crystallographic data and molecular dynamics simulations. In this way, we developed the first reported series of constrained peptide nuclear import inhibitors. Importantly, this general approach may be extended to other transcription factors.

The prognosis for ovarian clear cell carcinoma (CCC) patients with advanced‐stage disease is poor owing to intrinsic resistance to platinum‐based chemotherapy and the lack of targeted therapies available.^1^ Overexpression of the hepatocyte nuclear factor 1β (HNF1β) transcription factor is the most important clinical immunohistochemical marker for the disease, since it is ubiquitously overexpressed in CCC.^2^ However, to date, drugs targeting HNF1β have not been developed due to the high content of intrinsically disordered regions in transcription factors.^3^

Evidence that targeting HNF1β is a viable and attractive approach for developing a new targeted therapy was initially provided by Liu et al., who showed that downregulation of HNF1β increased cisplatin‐ and paclitaxel‐mediated cytotoxicity.^4^ HNF1β is expressed in the liver, digestive tract, pancreas, and kidneys, where it plays a role in early differentiation.^5^ Human HNF1β is made up of three domains: the dimerization domain; the transactivation domain, which is involved in binding transcriptional coactivators; and the DNA‐binding domain (DBD). We have recently confirmed the existence of a nuclear localization signal (NLS) within the DBD of HNF1β,^6^ which directs the nuclear import of the protein.^7^

Many NLS sequences are recognized in the cytoplasm by a heterodimeric transport carrier complex composed of importin α and importin β.^8^ Classical NLSs (cNLS) can bind to importin α through either a major site, a minor site, or both.^8^, ^9^ Monopartite cNLSs consist of a single cluster of positively charged residues, primarily lysines or arginines, that assume an ordered state once bound to importin α.^10^

Therapeutic targeting of the nuclear import of transcription factors provides a strategy for inhibiting their function, since activity depends on successful localization to the nucleus for transcription to take place.^11^ Lin et al. developed a 41‐residue synthetic peptide called cSN50 that contains the NF‐κB NLS and a cell‐permeable motif.^12^ The peptide inhibits the nuclear translocation of NF‐κB, attenuates gene transcription in intact cells, and is not cytotoxic within the concentration range of the experiments.^13^ cSN50 also inhibits the nuclear import of the transcription factors AP‐1, NFAT, and STAT1.^13^ However, it was readily digested during protease treatment with trypsin and pronase.^12^ cSN50 is the first nuclear‐import inhibitor that has shown importin α isoform specificity, binding with nanomolar affinity to importin α5 and only weakly to the other importin α isoforms.^14^ It also represents the first example of targeting the nuclear import of a transcription factor at the level of NLS recognition.^13^ To date, there has been no use reported of the technique of stapling^15^ to stabilize these intrinsically disordered NLS peptides. The aim of this work was to develop constrained peptide‐based inhibitors that target the HNF1β–importin α protein–protein interaction (PPI) and inhibit the activity of HNF1β. The proposed nuclear import targeting approach for the HNF1β–importin α PPI is summarized in Figure 1. The constrained peptide competes with HNF1β protein for importin α binding in the cytoplasm and is imported into the nucleus.

**Figure 1:**
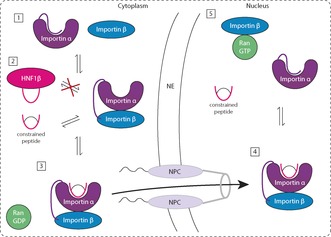
Proposed scheme for targeting the nuclear import of HNF1β through the HNF1β–importin α PPI: 1) The IBB domain of importin α binds to importin β to free up the NLS‐binding sites on importin α. 2) HNF1β NLS recognition by a heterodimeric complex composed of importin α and importin β, 3, 4) To enable HNF1β to be imported in the nucleus, the HNF1β NLS has to bind to the importin α–β heterodimer. The constrained peptide competes for this binding, thereby impairing the import of HNF1β. 5) Release of the constrained peptide through RanGTP binding to importin β. Reproduced and modified from Kobe et al.^16^

PPIs are crucial for many biological processes in the living cell and are responsible for the majority of cellular functions.^17^ Interestingly, it has been predicted that up to 49 % of transcription factor sequences are intrinsically disordered.^18^ Intrinsically disordered protein domains (IDD) do not assume well‐defined folded structures, but rapidly interconvert between different conformations.^19^ An example of IDDs are targeting motifs such as NLSs.^7^ Because IDDs have unique binding properties, conventional drug‐discovery strategies are less applicable for finding inhibitors, and novel strategies such as constrained‐peptide‐based approaches may be required.^19^, ^20^ Peptide‐based drugs are attractive alternatives to small‐molecule inhibitors owing to their high potency, specificity, and therapeutic safety.^21^ Compared to protein‐based drugs, they are less likely to initiate an immune response and their synthesis is more economical and less time consuming.^22^ Synthetic macrocylization through linking of the side chains of two non‐proteogenic amino acid residues allows peptides to be constrained in their bioactive conformation, thereby resulting in less entropy lost upon binding.^23^ In addition, the rate of proteolytic degradation of constrained peptides is often lower than that of their linear counterparts.^24^ Current challenges in the field include the design of cell‐permeable constrained peptides.

Our goal was to stabilize the HNF1β NLS peptide, which binds to importin α, in its binding conformation to give a constrained peptide with increased permeability whilst retaining potency. The crystal structure of the HNF1β NLS peptide bound to mImportin α1 ΔIBB (PDB ID: 5K9S), which has the autoinhibitory importin β binding (IBB) domain deleted, was used to aid the design of the constrained peptide.6c

We first performed cell proliferation experiments to validate the potential of HNF1β as a therapeutic target for the treatment of CCC. The effect on cell proliferation upon HNF1β knockdown was studied in five high‐ and low‐HNF1β‐expressing CCC cell lines and one high‐grade serous ovarian cancer (HGSOC) control cell line (PEO1), which does not express HNF1β. All of the CCC lines apart from JHOC7, OVISE, and PEO1 proliferated less upon small hairpin RNA (shRNA)‐mediated HNF1β knockdown (Figure 2). In the JHOC5, JHOC9, and SKOV3 cell lines, this reduction was found to be statistically significant, with *P*<0.02. These results are in agreement with previous results by Tomassetti et al. and Tsuchiya et al.,2b, 25 but we provide a more extensive investigation, with five cell lines and five time points. This work further validates HNF1β as a target for CCC.

**Figure 2:**
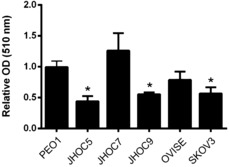
Relative proliferation of PEO1, JHOC5, JHOC7, JHOC9, OVISE, and SKOV3 CCC lines with *n*=4 after HNF1β shRNA knockdown. The mean is shown, with error bars showing the SEM. Statistical significance was assessed with multiple t‐tests and the Holm–Šídák method with *α*=5 %. Optical densities (ODs) are given relative to their respective non‐target knockdown OD value and background OD was subtracted. Only shRNA knockdown clone 583 at 96 h was considered here. * indicates *P*<0.02.

Because the binding affinity of cargo proteins for their carrier is an important factor for efficient nuclear import,^26^ it was imperative to quantify the dissociation constant (*K*_d_) of the HNF1β^DBD^ with its nuclear import protein (importin α) by isothermal titration calorimetry (ITC; see Figure S4 in the Supporting Information for ITC curve and binding parameters). The tighter binding (*K*_d_=625 nm) that was observed with the HNF1β^DBD^ protein compared to the much shorter HNF1β NLS peptide (*K*_d_=13.6 μm)6c can be rationalized by additional contributions to binding arising from the entire HNF1β^DBD^ protein.

Using a fluorescence polarization (FP) assay, the linear HNF1β NLS peptide sequence (Pep0: TAMRA‐5‐Ahx‐6‐TNKKMRRNRFK‐NH_2_) was determined to bind to mImportin α1 ΔIBB with a comparable *K*_d_ of 5.32 μm. This represents a roughly 2.5‐fold difference from that obtained previously by ITC (*K*_d_=13.6 μm).6c The FP assay was used for inhibitor testing.

To predict the most important binding interactions of the HNF1β NLS peptide with the mImportin α1 protein, molecular dynamics (MD) simulations for the complex were performed. Two key discrepancies between the crystal structure (PDB ID: 5K9S)6c and the MD simulations were identified. In the crystal structure, the backbone carbonyl of Thr1 from the peptide interacts with Arg238 of importin α (Figure 3 A), whereas in two out of three simulation runs, this interaction was lost and the side chain of Thr1 formed a hydrogen bond with Asp270 instead (Figure 3 B). Secondly, Arg9 of the peptide forms a salt bridge with Glu465 from a neighboring importin α chain in the crystal structure due to crystal packing (Figure 3 C), whereas it formed a salt bridge with Glu107 of importin α1 in all the simulation runs (Figure 3 D). These observations highlight both the influence of the crystal environment in inducing nonphysiological contacts and the importance of using MD simulations to eliminate the effect of crystal packing in the study of protein dynamics in solution.^27^ Our results suggest that both Thr1 and Arg9 should be retained to maintain the binding potency of the constrained peptides.

**Figure 3:**
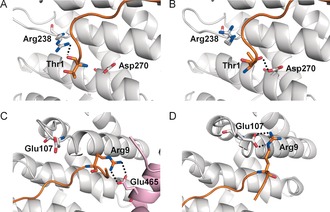
Binding interactions of HNF1β NLS peptide (orange) with mImportin α1 (gray) determined from X‐ray crystallography and MD simulations. The trajectory structures shown are final snapshots taken from the end of the simulations. A) The backbone carbonyl oxygen of Thr1 hydrogen bonds to the side chain of Arg238 in the obtained crystal structure (PDB ID: 5K9S). B) The side chain of Thr1 hydrogen bonds with the side chain of Asp270 in the MD simulations. C) Arg9 forms a salt bridge with Glu465 from a neighboring protein chain (pink) in the crystal structure. D) Arg9 forms a salt bridge with Glu107 in the MD simulations.

The contribution of each HNF1β NLS residue to the binding was then assessed by binding free energy decomposition,^28^ based on the structures of HNF1β NLS peptide in complex with mImportin α1 obtained from the MD simulations. Residues Asn2, Asn8, Phe10, and Lys11 contributed very little to the total binding free energy, thus suggesting that they could be removed from the peptide with minimal disruption to the overall binding (Figure 4 A). Computational alanine scanning was then used to determine suitable stapling locations. Each peptide residue was mutated to alanine and the difference in the binding free energy between the mutant and wild‐type complexes calculated (Figure 4 B). The results were in agreement with the binding free energy decomposition analysis (Figure 4 A). Thr1, Lys4, Arg6, Arg7, and Arg9 were identified as the most important residues for binding, whereas Asn2, Asn8, Phe10, and Lys11 made only negligible contributions to the binding. Both energetic analyses indicate that the constrained peptide inhibitors should be designed based on the following peptide sequence: ^1^TNKKMRRNR^9^. Since the constraining linker should preferably be placed on residues where the side chains have little or negative contribution to the binding,^29^ residues Asn2 and Asn8 were chosen for replacement with a linker. The constraints were introduced by using unnatural azido amino acids and dialkynyl linkers through two‐component double‐click chemistry (Figure 5).^30^

**Figure 4:**
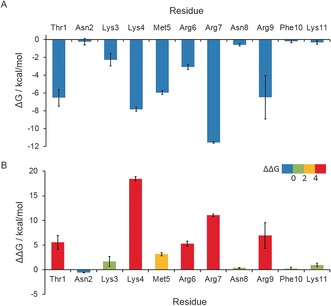
Energetic analysis of the MD simulations of the complex of HNF1β NLS peptide with mImportin α1. A) Binding free energy contributions of HNF1β NLS peptide residues. B) Computational alanine scanning of HNF1β NLS peptide residues. Hot, warm, cool, and cold spots are shown in red, orange, green, and blue, respectively.

**Figure 5:**
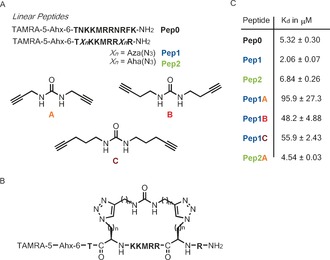
A) Synthesized peptide sequences containing azido amino acids and linkers A–C. B) General structure of the bis‐triazole‐constrained peptides with *n*=1,2 and *m*=1–3. C) Direct FP assay binding affinities for (constrained) peptides in μm. The full synthesis of the intermediates and constrained peptides can be found in the Supporting Information.

The unconstrained peptides Pep1 and Pep2, as well as four constrained peptides, were synthesized. Their binding affinities for mImportin α1 ΔIBB were evaluated in fluorescence polarization assays (Figure 5 C). The introduction of the unnatural azido amino acids did not have an adverse effect on the binding of the peptides. Compared to the linear wild‐type peptide Pep0, there was a 2.5‐fold improvement in the binding of Pep1, while Pep2 exhibited slightly decreased binding potency. The constrained peptides Pep1A–Pep1C followed a rough trend in which binding affinities increased with increasing linker length (Figure 5). However, their binding affinities were still about an order of magnitude weaker than that of Pep0. The linkers were possibly too short to constrain these peptides in the appropriate conformation for binding, thereby resulting in higher *K*_d_ values. In contrast, Pep2A (*K*_d_=4.54 μm, Figure 6), which is formed from unnatural azido amino acids with longer side chains, was observed to bind with slightly improved binding affinity compared to Pep0. This highlights the importance of optimizing side‐chain lengths in the synthesis of “double‐click” peptides.^31^ Significantly, this is the first time that a constrained HNF1β NLS peptide has been found to bind more tightly than its unconstrained peptide precursor.

**Figure 6:**
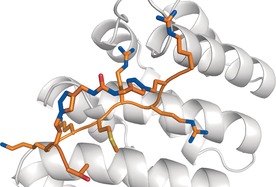
A model of the constrained peptide Pep2A (orange) bound to mImportin α1 ΔIBB (gray).

Live‐cell fluorescence microscopy studies were undertaken to assess the cell permeability of the synthesized TAMRA‐labelled linear and constrained peptides (Figure 7). Pep0 showed limited cell permeability, while Pep1 and in particular Pep2 showed good cell permeability. The corresponding constrained peptides Pep1B and Pep2A retained their cell permeability upon stabilization. This work represents the first example of constraining an NLS peptide to target the nuclear import pathway, and it does not require the attachment of a cell‐permeable peptide sequence. All of the constrained peptides were more cell‐permeable than the linear HNF1β NLS control peptide Pep0.

**Figure 7:**
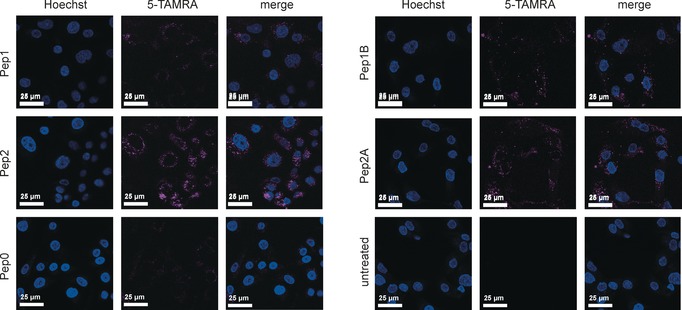
Cell‐permeability studies for the linear and constrained peptides using JHOC9 cells. Images were taken on a Leica tandem confocal microscope using a 40 X objective.

In conclusion, we have further validated HNF1β as a therapeutic target in CCC by including more CCC lines and time points during knockdown studies than previous efforts.2a,2b A set of constrained peptide inhibitors based on the HNF1β NLS sequence was developed using rational drug design to competitively target the HNF1β–importin α PPI, and binding data were obtained using both ITC and FP assays. MD simulations were performed to guide the development of constrained peptides that have enhanced conformational similarity to the bound HNF1β NLS peptide, thus further reducing the entropic penalty for binding.^29^ A constrained peptide, Pep2A, which had a higher binding affinity than that of the unconstrained HNF1β NLS peptide Pep0, and which bound more tightly than its unconstrained precursor Pep2, was identified. This confirmed that an entropically‐driven gain in binding affinity was achieved for Pep2A. All of the constrained peptides, including Pep2A, were more cell‐permeable than Pep0. This work provides the first example of using constrained peptides that mimic the ordered state of NLSs to target the nuclear import of transcription factors. Further studies are now underway to elucidate the structural conformation of the constrained peptides upon binding to the target protein. The surrounding residues of an NLS are often important for binding specificity.^32^ For example, the transcription factor Stat1 has been found to be specifically imported by importin α5 both in vitro and in vivo, and this specificity appears to rely on contacts made with the C‐terminal acidic region of importin α5.^33^ Further structural information on the binding of HNF1β to importin α is required for the future design of isoform‐selective importin α inhibitors. HNF1β overexpression in breast cancer^34^ and pancreatic clear cell carcinoma^35^ also correlates with worse survival rates, and the developed constrained peptide inhibitors may have a therapeutic effect on breast, pancreatic, and ovarian clear cell carcinoma proliferation.^36^ This method of rational design of constrained‐peptide drug candidates should also be applicable to other IDDs.

*Dedicated to Professor Stuart L. Schreiber on the occasion of his 60th birthday*

## Supporting information

As a service to our authors and readers, this journal provides supporting information supplied by the authors. Such materials are peer reviewed and may be re‐organized for online delivery, but are not copy‐edited or typeset. Technical support issues arising from supporting information (other than missing files) should be addressed to the authors.

Supplementary
