# Supplementary material for: Development of Cell‐Permeable, Non‐Helical Constrained Peptides to Target a Key Protein–Protein Interaction in Ovarian Cancer
Source: Angew Chem Int Ed Engl. 2016 Dec 5;56(2):524–9. doi: 10.1002/anie.201609427 (PMC5291322; doi:10.1002/anie.201609427)
Supplement: Supplementary file 1 — Supplementary [file ANIE-56-524-s001.pdf]

Angewandte Chemie
Eine Zeitschrift der Gesellschaft Deutscher Chemiker

Supporting Information

Development of Cell-Permeable, Non-Helical Constrained Peptides to Target a Key Protein–Protein Interaction in Ovarian Cancer

*Mareike M. Wiedmann, Yaw Sing Tan, Yuteng Wu, Shintaro Aibara, Wenshu Xu, Hannah F. Sore, Chandra S. Verma, Laura Itzhaki, Murray Stewart, James D. Brenton, and David R. Spring**

anie_201609427_sm_miscellaneous_information.pdf

Supporting Information

METHODS 2

MAMMALIAN CELL CULTURE 2
PROTEIN EXTRACTION FROM MAMMALIAN CELLS 2
WESTERN BLOTTING 2
CELL PERMEABILITY ASSAY AND CONFOCAL MICROSCOPY 2
LENTIVIRUS PRODUCTION AND TRANSDUCTION 2
HNF1B PROTEIN KNOCKDOWN LEVELS – PROLIFERATION ASSAY 3
HNF1B PROTEIN KNOCKDOWN LEVELS – WESTERN BLOTS 5
SULFORHODAMINE B ASSAY 5
GENE CLONING OF HNF1B DNA-BINDING DOMAIN 5
PROTEIN EXPRESSION IN BACTERIAL CELLS AND PURIFICATION 5
ISOTHERMAL CALORIMETRY EXPERIMENTS 5
MOLECULAR DYNAMICS SIMULATIONS 6
BINDING FREE ENERGY DECOMPOSITION 7
COMPUTATIONAL ALANINE SCANNING 7
AZIDO AMINO ACID SYNTHESIS 7
TAMRA-5 SYNTHESIS 7
PEPTIDE SYNTHESIS BY FMOC-SOLID PHASE PEPTIDE SYNTHESIS 8
DIALKYNE LINKER SYNTHESIS 8
MANUAL FMOC-SOLID PHASE PEPTIDE SYNTHESIS 8
AUTOMATED FMOC-SOLID PHASE PEPTIDE SYNTHESIS 9
CONSTRAINED PEPTIDES SYNTHESIS BY COPPER DOUBLE-CLICK REACTION 9
DIRECT FLUORESCENCE POLARISATION ASSAY 9
GENERAL CHEMISTRY EXPERIMENTAL 11
SYNTHESIS OF AZIDO AMINO ACIDS 12
FMOC-AZA-OH (1) 12
FMOC-AHA-OH (2) 12
IMIDAZOLE-1-SULFONYL AZIDE HYDROGEN SULFATE (7) 13
SYNTHESIS OF DIALKYNYL LINKERS 13
1,3-DI(PROP-2-YN-1-YL)UREA (13) 13
1,3-DI(BUT-3-YN-1-YL)UREA (14) 13
1,3-DI(PENT-4-YN-1-YL)UREA (15) 14
SYNTHESIS OF TAMRA-5 14
4-(4-(DIMETHYLAMINO)-2-HYDROXYBENZOYL)ISOPHTHALIC ACID (11) 14
5-CARBOXYTETRAMETHYLRHODAMINE (TAMRA-5) (8) 14

^{1}H AND ^{13}C NMR SPECTRA 16

PEPTIDE MASS SPECTROMETRY CHARACTERISATION DATA 24

ANALYTICAL PEPTIDE TRACES 25

UNCONSTRAINED PEPTIDES MASS SPECTROMETRY TRACES 25
CONSTRAINED PEPTIDES MASS SPECTROMETRY TRACES 26

REFERENCES 27

Methods

Mammalian cell culture

HEK293T cells were cultivated in Dulbecco's Modified Eagle medium (DMEM) (1X) supplemented with 5 % foetal bovine serum (FBS) (Invitrogen) and 0.5 % penicillin/streptomycin (P/S). CCC cell lines PEO1, JHOC5, JHOC7, JHOC9, OVISE, SKOV3 and Normal Ovarian Surface Epithelial (IOSE) cells were grown in RPMI 1640 medium (1X) supplemented with 10 % FBS and 1 % P/S. All cell lines were maintained at 37 °C in 5 % CO_2 and were mycoplasma tested on a regular basis (Biorepository Core, CRUK CI, Cambridge). Cell counts were conducted using the Vi-CELL Cell Viability Analyzer.

Protein extraction from mammalian cells

Cell pellets were washed with phosphate buffered saline (PBS) and 200 µL protein lysis buffer (50 mM Tris pH 8.0, 150 mM NaCl, 5 mM EDTA, 0.5 % Igepal, 2 tablets/100 mL protease inhibitor cocktail tablet (Roche)) was added. The mixtures were incubated on ice for 30 min, lysed by syringing four times using a 26 G needle and centrifuged at 14,800 × g for 10 min at 4 °C. Protein concentrations were measured using the DirectDetect IR spectrometer (Merck Millipore) according to the manufacturer's instructions.

Western blotting

Denatured protein extracts were separated using NuPAGE Novex 4-20 % Tris-Glycine Gel and transferred to a Millipore Immobilon FL PVDF membrane (Invitrogen). Primary antibodies were used as follows: anti-HNF1β (goat) (sc-7411, polyclonal, Santa Cruz Biotechnology, 1:1,000) and anti-GAPDH (rabbit) (G9545, 14C10, 1:5,000, Cell Signaling Technology). Odyssey Infrared Imaging System (Li-Cor) and associated secondary antibodies: donkey anti-goat (800) (1:15,000) and donkey anti-rabbit (800) (1:5,000) were used to detect protein.

Cell permeability assay and confocal microscopy

Cells were grown in chambered coverslips (µ–Slide 8 Well ibiTreat, Ibidi) for live-cell imaging. When the desired degree of confluency was reached, the medium was replaced with 10 µM solutions of constrained peptides in medium. For live-cell imaging, cells were incubated with the constrained peptide solutions for 2 h and during the final 30 min, Hoechst stain 33342 (1 µM, Thermo Fisher Scientific) was added. Cells were washed with PBS before live-cell imaging. Fresh medium was added and cells were imaged live using a Leica Tandem confocal microscope. Images were generated as a maximum intensity projection of five z-stacked images.

Lentivirus production and transduction

A general protocol by Cribbs *et al*. was used for lentivirus production.[1] Four different knockdown clones termed 577, 578, 582 and 583 (Sigma Aldrich, TRC2-pLKO-puro Vector, TRCN0000255<u>577</u>, TRCN0000255<u>578</u>, TRCN0000255<u>582</u>, TRCN0000255<u>583</u>) and a non-target shRNA control SHC202 (Sigma Aldrich, Mission RNAi, TRC2-pLKO-puro Vector) were used. All plasmids were confirmed by restriction digest and agarose gel electrophoresis.

For each transfection sample 16 µg transfer vector, 10.4 µg pMDL/pRRE, 4 µg pRSV-Rev and 5.6 µg pVSV-G were used. Lentiviruses were produced in the HEK293T packaging cell line. The day before transfection, 16 x 10^6 HEK293T cells were plated in 175 mL flasks (Corning) using Opti-MEM reduced-serum medium (30 mL, 1 X, Invitrogen) so that cells were 90–95 % confluent on the day of transfection. After 24 h the medium was removed and replaced with DMEM growth medium (16 mL, Invitrogen) containing serum. For each transfection sample DNA-Lipofectamine complexes were prepared as follows: In a sterile 15 mL tube, 16 µg transfer vector, 10.4 µg pMDL/pRRE, 4 µg pRSV-REV and 5.6 µg pVSV-G were diluted in Opti-MEM medium (5 mL). In a separate sterile 15 mL tube, Lipofectamine 2000 (115 µL, Life Technologies) was diluted in Opti-MEM medium (5 mL). Both solutions were incubated for 5 min at room temperature and then combined. Incubation for 20 min at room temperature allowed DNA-Lipofectamine complexes to form, which were then added dropwise to each plate of HEK293T cells. Cells were incubated overnight at 37 °C in a humidified 5 % CO_2 incubator. After 24 h, expression of GFP protein was observed in the GFP control virus HEK293T sample.

Virus containing supernatant was harvested posttransfection according to the protocol by Kutner *et al*.[2] Virus-containing supernatant was harvested 48–72 h post-transfection by transferring the cell culture medium into 15 mL sterile tubes. The supernatant was centrifuged at 3,000 rpm for 15 min at 4 ◦C to pellet debris and filtered through a Millex-HV 0.45 µm PVDF filter. The resulting supernatant was stored at 4 °C. 30 mL centrifuge tubes (Beckman Coulter Conical) were sterilised by spraying with 70 % EtOH and placed in SW28 buckets on top of their specific adaptors. Virus-containing supernatant (22 mL) was added and topped up to approximately 2–3 mm from the top with PBS. The solutions were ultracentrifuged using a SW28 rotor for 2 h at 4 °C at 21,400 rpm. The supernatant was drained and discarded into 2 % Virkon (Chemours), and residual droplets were aspirated. Tubes were placed in sterile 50 mL falcon tubes and 100 µL PBS was added to each viral pellet. The pellets were resuspended for 24 h with gentle agitation on a rocker at 4 °C. Concentrated viral solutions were resuspended and transferred to cryovials in 10 µL aliquots and stored at -80 °C until use.

A functional method was used to determine the titre. Analysis of the number of transducing units (TU) by flow cytometry analysis of a reporter protein (GFP) was conducted. Lentiviral stocks were titred using flow cytometry analysis. This method does not permit discrimination between single and multiple viral integrations and to counteract, only transduced cells that showed a less than 40 % GFP-positivity were analysed.[3] For titration, 1 x 10^5 cells in 500 µL DMEM medium with 5 % FBS per well were seeded in a 12-well plate (Corning). Virus-containing solutions were pipetted over the HEK293T cells. Duplicate virus dilutions of 1:500, 1:1000, 1:2000, 1:5000 were added. An untreated control was included. The medium was changed 24 h post-transfection and cells were harvested after 72 h by trypsinisation in the usual way. Cells were collected by centrifugation at 500 g for 5 min at room temperature. The supernatant was discarded and cell pellets were resuspended in PBS. A GFP-propidium iodide (GFP-PI) based assay for flow cytometric measurement of transfection efficiency and cell viability was performed on an LSR-II machine (BD). The titre was calculated from dilutions that gave 1–40 % GFP-positivity and averaged subsequently using the following formula:

$$\text{Titre}\left(\frac{\text{TU}}{\text{mL}}\right) = \frac{(\text{frequency of GPF-positive cells}) \times \text{no. cells plated} \times \text{dilution factor}}{\text{volume of inoculum}}$$

where, frequency of GFP-positive cells is the percentage of cells that are GFP-positive divided by 100 (acceptable range: 0.01–0.40), dilution factor is the dilution of the viral stock used and volume of inoculum is the total volume transduced. The titre was calculated to be 2.49 x 10^8 TU/mL for all shRNA knockdown clones.

For each cell line 1 x 10^6 cells in 500 µL medium per well were seeded in a 12-well plate (Corning). Virus-containing solutions were added so that the desired multiplicities of infection (MOI) were obtained. Polybrene 0.5-10 µg/mL was added to increase transduction efficiency. Transduced cells were harvested and knockdown levels were determined 24 and 72 h post-transduction by western blot analysis. Transduction efficiencies were determined by flow cytometry analysis using an LSR-II machine (BD).

HNF1β protein knockdown levels – Proliferation assay

For shRNA knockdown experiments using a lentiviral vector cell lines OVISE, SKOV3, JHOC5, JHOC7 and JHOC9 were used as models of CCC. As a negative control, high grade serous ovarian carcinoma (HGSOC) line PEO1 was used, which does not express HNF1β. The difference in proliferation of cell lines transduced with shRNA knockdown clone 583 and non-target shRNA clone SHC202 was plotted (Figure 1 in paper). Four shRNA knockdown clones 577, 578, 582, 583 and non-target shRNA SHC202 were used in experiments to study the effect of HNF1β knockdown in CCC lines. Identification of the most effective shRNA knockdown clone was evaluated in JHOC5 and JHCO9 lines (Figure 1). An MOI of 1 was used in all experiments.
Highest protein knockdown levels were achieved using shRNA knockdown clone 583 in both cell lines (B, D). Clone 583 also gave the greatest reduction in proliferation (Figure 1A and Figure 1C). Therefore only shRNA knockdown clone 583 was used in further knockdown experiments using JHOC7, OVISE, SKOV3 and PEO1 line (Figure 2).
A virus-related reduction in proliferation was observed in some cell lines (Figure 2A and Figure 2B). As evident from Figure 2A, HGSOC line PEO1 was not affected by HNF1β shRNA 583 knockdown apart from virus-related toxicity effects and HNF1β protein expression was not detected (Figure 3). The identical drop in proliferation was observed using non-target shRNA clone SHC202. CCC lines OVISE and SKOV3 featured a reduced proliferation upon HNF1β shRNA 583 knockdown and only CCC line JHOC7 was unaffected (Figure 2). Western blot data for all proliferation experiments is displayed in Figure 3.

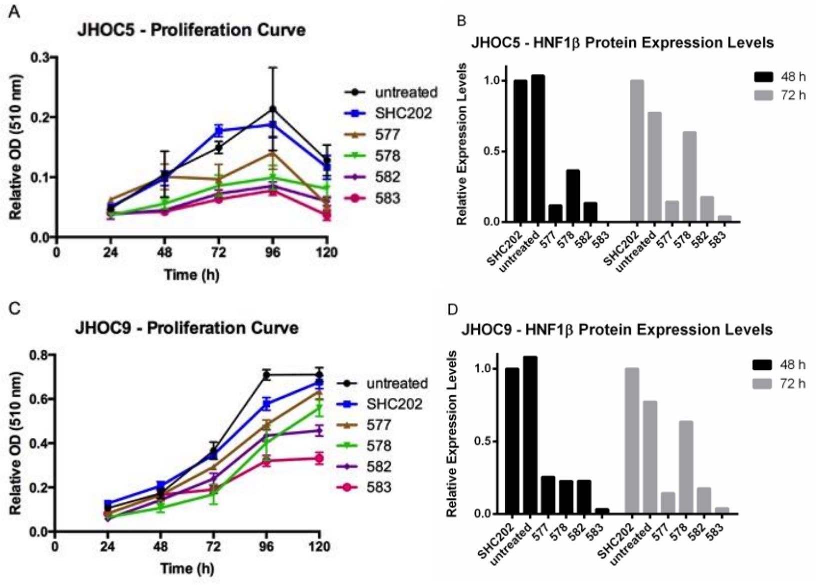

Figure 1. HNF1β knockdown in JHOC5 and JHOC9 lines using shRNA knockdown clones 577, 578, 582, 583 and non-target control SHC202. For the SRB proliferation assay (A, C) three biological and four technical replicates were included. Data is the mean and SEM. HNF1β protein expression levels were determined 48 and 72 h post-transduction (B, D) and were normalised to the non-target SHC202 protein expression levels using GAPDH as the loading control.

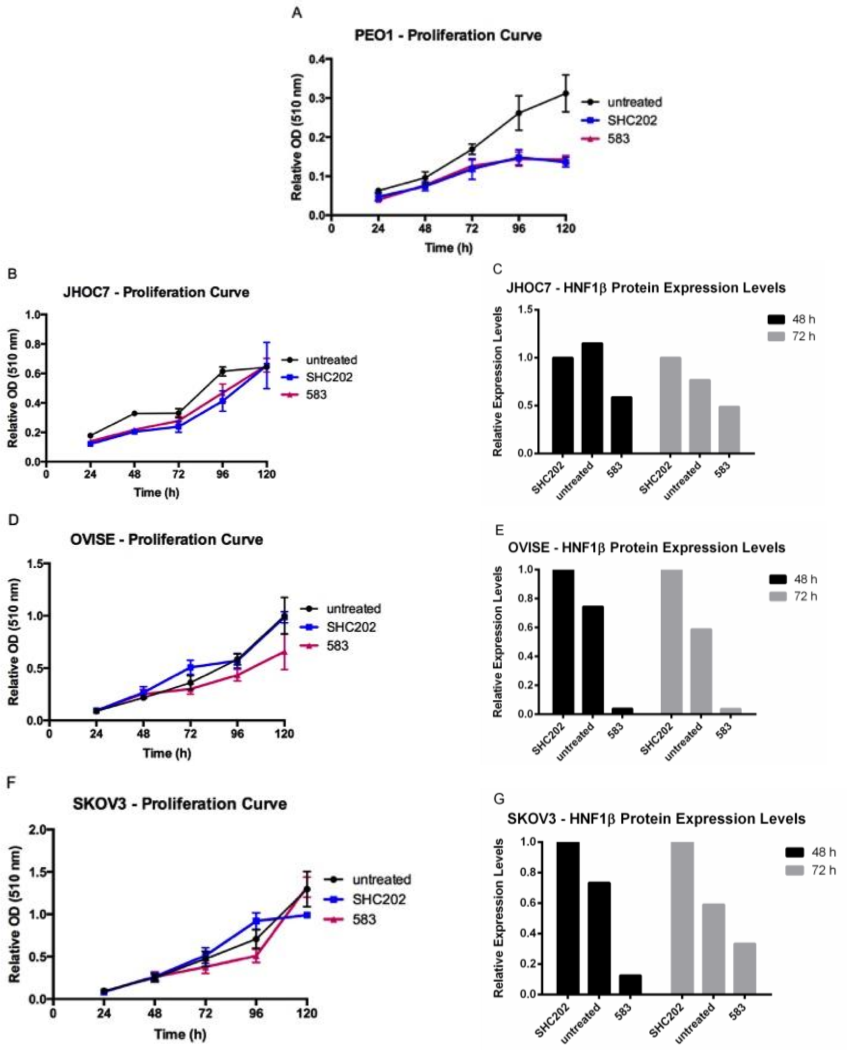

Figure 2. HNF1β knockdown in PEO1, JHOC7, OVISE and SKOV3 lines using shRNA knockdown clone 583 and non-target control SHC202. For the SRB proliferation assay (A, B, D, F) three biological and four technical replicates were included. Data is the mean and SEM. HNF1β protein expression levels were determined 48 and 72 h post-transduction (C, E, G) and were normalised to SHC202 protein expression levels using GAPDH as the loading control.

HNF1β protein knockdown levels – Western Blots

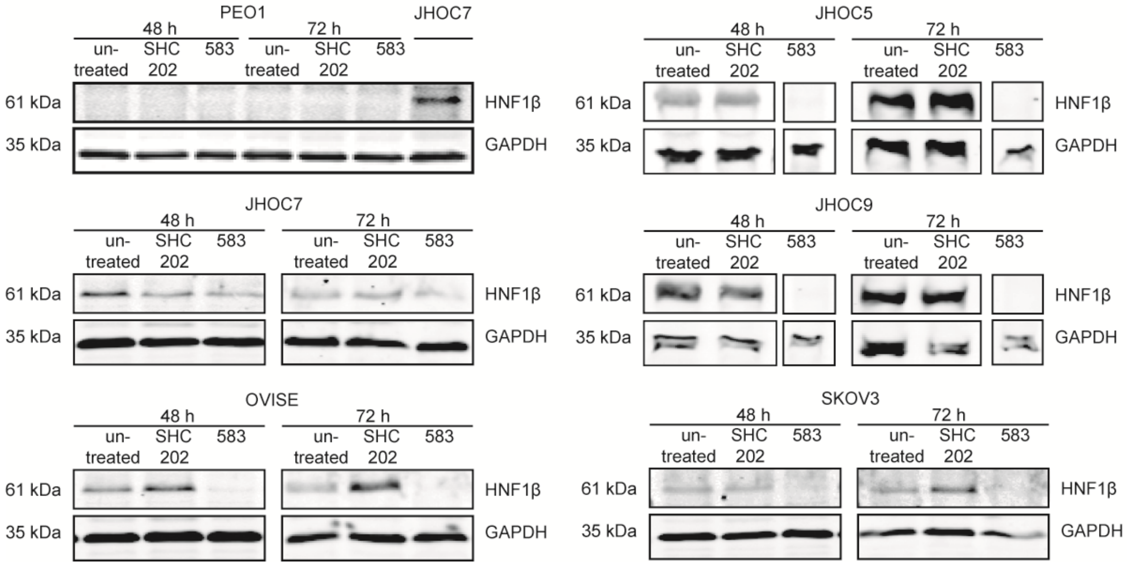

Figure 3. HNF1β protein expression levels 48 and 72 h post-transduction with shRNA knockdown clone 583 and shRNA non-target control SHC202 in PEO1, JHOC5, JHOC7, JHOC9, OVISE and SKOV3 lines. For HGSOC line PEO1, JHOC7 protein lysates were used as a positive staining control. GAPDH was used as the loading control.

Sulforhodamine B Assay

Cells were plated in a 96-well plate (Corning) and incubated overnight. At each time point the medium was removed and 100 µL trichloroacetic acid (TCA) was added. Cells were fixed at 4 °C overnight. TCA was removed and plates were washed four times with water. Plates were allowed to dry and were subsequently stained with Sulforhodamine B (SRB) stain for 30 min at room temperature. Plates were washed four times with 1 % AcOH using a plate washer (BioTek ELX405), dried and resuspended in 200 µL Tris buffer (pH 10) on a shaker for 10 min. Optical densities (ODs) were read on a PHERAstar FS (BMG Labtech) at 510 nm. Wells containing only medium were used as a background control.

Gene cloning of HNF1β DNA-binding domain

The DNA-binding domain of HNF1β (HNF1β^{DBD}) (91-310) was cloned into a pGEX-TEV vector. The plasmid was confirmed by sequencing by GATC (Konstanz, Germany).

Protein expression in bacterial cells and purification

All proteins were expressed in *E. coli* BL21 (DE3) CodonPlus-RIL cells using IPTG induction over 18 h at 18 °C. The cells were harvested by centrifugation and resuspended in 50 mM Tris/HCl pH 8.0, 500 mM NaCl, 5 mM DTT for GST-tagged constructs or 50 mM Tris/HCl pH 8.0, 500 mM NaCl, 20 mM Imidazole pH 8.0 for His_6-tagged proteins. The *E. coli* were lysed by two passes through an Emulsiflex C3 system (AVESTIN) running at a pressure of 15,000 psi. The lysates were clarified by centrifugation at 48,000 × g. Either Ni-NTA agarose resin (QIAGEN) or glutathione sepharose 4B resin (GE Healthcare) was added to the clarified lysate containing His_6-tagged proteins or GST-tagged proteins. The resin was pooled and packed into a gravity filtration column and washed extensively with their respective lysis buffers. 1 mg of TEV protease (purified in-house) was added to the washed resin and incubated for 18 h at 4 °C. The tag-free protein of interest was then collected from the flow-through and subjected to the next purification step. HNF1β^{DBD} was purified further by heparin affinity chromatography using a HiTrap Heparin HP column (GE Healthcare Life Sciences). HNF1β^{DBD} was eluted from the column by running a gradient between 100 mM and 1 M NaCl. All proteins were purified to homogeneity by size exclusion chromatography using a HiLoad™ 26/60 Superdex™ 75 prep grade column (GE Healthcare) pre-equilibrated in 20 mM HEPES pH 8.0, 200 mM NaCl. Fractions containing pure and homogenous proteins identified by SDS-PAGE analysis were pooled and concentrated using an Amicon Ultra 15 centrifugation filtration unit. Protein concentrations were measured by UV-Vis spectroscopy using a Nanodrop (Thermo) and small aliquots of the protein were flash frozen in liquid nitrogen and stored at -80 °C until required.

Isothermal calorimetry experiments

The K_d of mImportin α1 with the HNF1β^{DBD} was determined by isothermal titration calorimetry (ITC) using a MicroCal™ iTC200 (GE Healthcare Life Sciences). The protein samples were made up in identical buffer (20 mM Hepes (pH 8), 200 mM NaCl).

350 µL of protein sample (0.02 mM) and 200 µL of ligand (0.2 mM) were prepared for each experiment. Blank experiments were performed by titrating ligand into buffer. Sample concentrations were confirmed by amino acid analysis (PNAC Facility, Biochemistry Department, Cambridge University). All experiments were conducted at 25 °C. The data was analysed using the Origin™ Software (MicroCal) using a one site binding model.

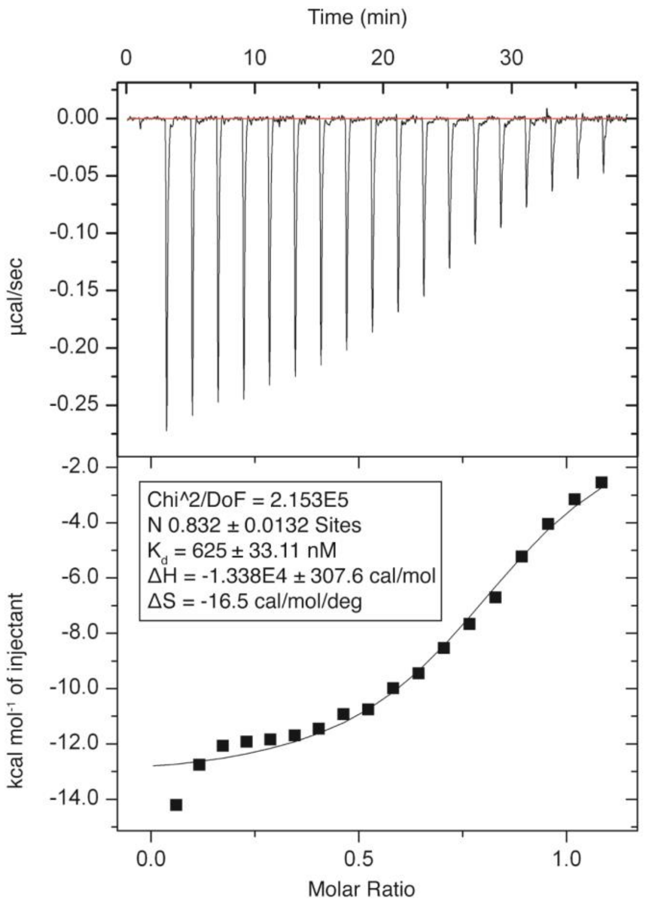

Figure 4. Dilution-corrected ITC curve and integrated heat data of the mImportin α1 ΔIBB interaction with HNF1β^{DBD} protein.

Molecular dynamics simulations

The structure of mImportin α1 in complex with HNF1β NLS peptide (^{1}TNKKMRRNRFK^{11}) (PDB: 5K9S [4]) was used as the initial structure for molecular dynamics (MD) simulations. The HNF1β NLS peptide bound at the minor site was removed while the peptide bound at the major site was retained. Importin α1 was capped at its N- and C-termini by acetyl and N-methyl groups respectively while the HNF1β NLS peptide was capped at its N- and C-termini by acetyl and amide groups respectively. Crystallographic water molecules were retained. PDB2PQR [5] was then used to determine protonation states of residues. Using the LEaP module in the AMBER 14 [6] package, the complex structure was solvated with TIP3P water molecules in a periodic truncated octahedron box such that there is a minimal distance of 10 Å between the box walls and complex, followed by neutralisation of the system with sodium ions.[7]

Energy minimisations and MD simulations were carried out by the PMEMD module of AMBER 14 using the ff14SB force field.[8] A total of three independent explicit-solvent MD simulations using different initial atomic velocities were carried out. The SHAKE algorithm [9] was applied to constrain all bonds involving hydrogen atoms, allowing for a time step of 2 fs. A cutoff distance of 9 Å was implemented for nonbonded interactions. The particle mesh Ewald method [10] was used to treat electrostatic interactions with a grid spacing of 1.0 Å and the order of B-spline interpolation set to 4. Energy minimisation was performed for 500 steps with the steepest descent algorithm, followed by another 500 steps with the conjugate gradient algorithm. The system was then heated gradually to 300 K over 50 ps at constant volume before equilibration at a constant pressure of 1 atm for another 50 ps. During minimisation and equilibration, weak harmonic positional restraints with a force constant of 2.0 kcal mol^{-1} Å^{-2} were imposed on the non-hydrogen atoms of the solute. These restraints were removed in a subsequent equilibration (2 ns) run and the production (100 ns) run, which were carried out at 300 K and 1 atm. The Langevin thermostat [11] was used to maintain the temperature with a collision frequency of 2 ps^{-1}. Pressure was maintained by a Berendsen barostat [12] with a pressure relaxation time of 2 ps.

Binding free energy decomposition

The contribution of each HNF1β NLS peptide residue to the binding free energy of complex formation was computed by applying the free energy decomposition method [13] on 200 equally-spaced structures extracted from the last 40 ns of the MD simulations of the HNF1β–Importin α1 complex. Binding free energies were calculated in AMBER 14 [6] using the molecular mechanics/generalised Born surface area (MM/GBSA) method.[14] The molecular mechanical energies and polar contribution to solvation free energy were computed by the sander module and pbsa program using the modified GB model described by Onufriev *et al*. respectively.[15] The nonpolar contribution to solvation free energy was estimated from the solvent accessible surface area (SASA) using the ICOSA method.[16] Free energy values are represented in Figure 3A as an average of the values obtained from each of the three simulation runs.

Computational alanine scanning

Computational alanine scanning of each HNF1β NLS peptide residue was carried out on 200 equally-spaced structures extracted from the last 40 ns of the MD simulations of the HNF1β–Importin α1 complex. The difference in the binding free energies ($\Delta\Delta G_{bind}$) of the wild type and alanine mutants (or glycine mutants for mutated alanine residues) of the peptide was calculated using the MM/GBSA method.[14]

$$\Delta\Delta G_{bind} = \Delta G_{mutant} - \Delta G_{wild\ type} \qquad (1)$$

Similar to the binding free energy decomposition, binding free energies were calculated using modules in AMBER 14.[6] Molecular mechanical energies were calculated with the sander module. The polar contribution to the solvation free energy was calculated by the pbsa program [17] in AMBER 14 using the modified GB model described by Onufriev *et al.* [15] while the nonpolar contribution was estimated from the SASA using the linear combinations of pairwise overlaps method,[18] with γ set to 0.0072 kcal mol^{-1} $Å^{-2}$ and β to zero.[19] The entropy term was not considered due to the high computational cost and the assumption that the entropy of the mutant does not differ considerably from that of the wild type.[20] Free energy values are represented in Figure 3B in the paper as an average of the values obtained from each of the three simulation runs.

Azido Amino Acid Synthesis

Azido amino acids **1** and **2** required for peptide stapling were synthesised according to **Scheme 1** from Fmoc-protected starting materials Fmoc-Asn-OH **3** and Fmoc-Gln-OH **4**. Hofmann rearrangement with [bis(trifloroacetoxy)iodo]benzene gave Fmoc-Dap-OH **5** and Fmoc-Dab-OH **6** followed by an azide transfer reaction [21] with **7** gave azido amino acids Fmoc-Aza-OH **1** and Fmoc-Aha-OH **2** in overall acceptable yield over two steps.

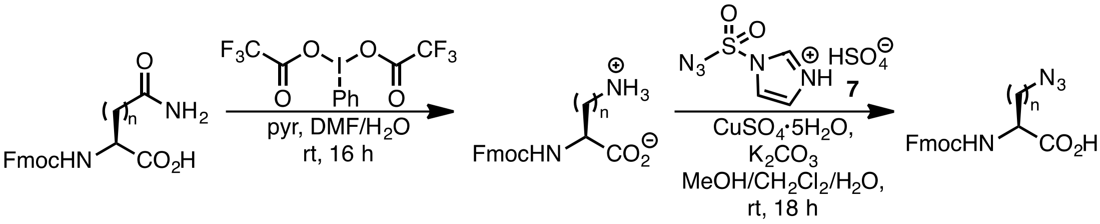

3 (n = 1) **5** (n = 1) **1** (n = 1) 48%
4 (n = 2) **6** (n = 2) **2** (n = 2) 35%

Scheme 1. Synthesis of azido amino acids **1** and **2** by Hoffmann rearrangement and azide transfer reaction

The azide transfer reagent 9 required in the second step (**Scheme 1**) was obtained in 81% yield from sulfuryl chloride, imidazole and sodium azide (**Scheme 2**). Due to safety concerns, the hydrogen sulfate salt was chosen over the equivalent hydrogen chloride salt of the azide transfer reagent since it is less shock sensitive and has a higher decomposition temperature (131 °C rather than 102 °C). The NMR data of azide transfer reagent **7** revealed additional peaks probably arising from the spontaneous cleavage of the azido sulfonyl moiety leaving an imidazolium salt.[22]

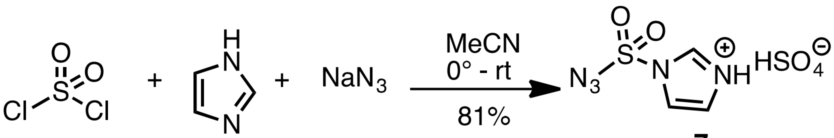

7

Scheme 2. Synthesis of azide transfer reagent **7**

TAMRA-5 Synthesis

TAMRA dyes have been used in the literature to label DNA,[23] oligonucleotides [24] or peptides [25] and have also been used extensively to study DNA-protein interactions.[26] TAMRA-5 **8** was synthesised (**Scheme 3**) according to the procedure of Kvach *et al.*[27] Nucleophilic addition of 3-dimethylamino phenol **9** into trimellitic anhydride **10** gave intermediate **11** in 15% yield. The low yield was attributed to the multiple washes with

MeOH required to remove isomer **12** (precursor to TAMRA-6), in order to give pure TAMRA-5 precursor **11**. TAMRA-5 **8** was obtained by a second nucleophilic addition of 3-dimethylamino phenol **9** followed by a double-dehydration while refluxing at 110 °C in DMF. Trimethylsilylpolyphosphate was used as the dehydrating agent and weak acid catalyst during TAMRA-5 synthesis.[28] The fluorescent dye, TAMRA-5 **8**, was later coupled to the synthesised peptides to allow measurement of binding constants between the synthesised inhibitors and Importin α1.

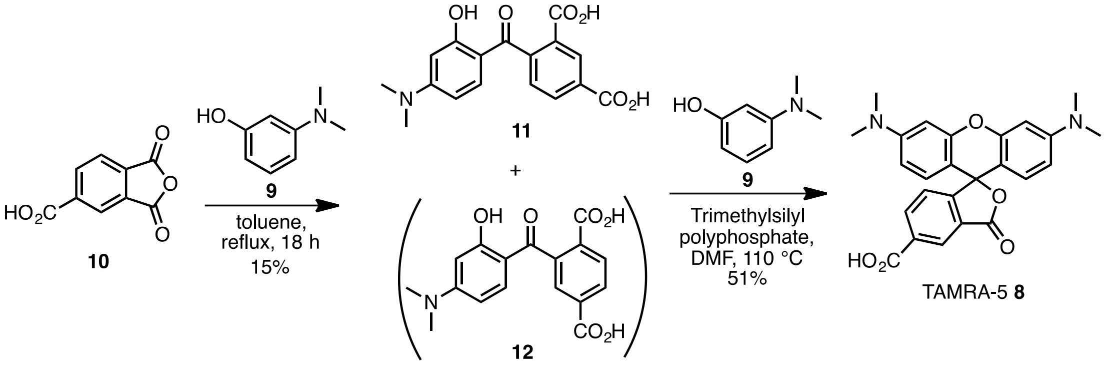

Scheme 3. TAMRA-5 **8** synthesis according to the procedure of Kvach *et al.* [27]

Peptide Synthesis by Fmoc-Solid Phase Peptide Synthesis

Peptides were synthesised (0.10 mmol scale) using Fmoc-Solid Phase Peptide Synthesis (Fmoc-SPPS). This method was preferred over the alternative Boc-SPPS as the former requires milder cleavage reagents such as TFA as opposed to hazardous hydrogen fluoride.[29] Fmoc-protecting groups were removed with piperidine in DMF. For amino acid couplings HATU and DIPEA were used. Completeness of coupling reactions was assessed using the Chloranil test.[30] The previously synthesised azido amino acids were incorporated into the peptide for later stapling. Insertion of a short spacer amino acid in between the stapled peptide and the fluorescent dye was thought beneficial to minimise steric interactions of the dye with the binding motif. Hence Ahx-6-OH was inserted before peptides were capped with TAMRA-5. Peptides were cleaved off the resin with simultaneous side-chain deprotection using a cleavage mixture consisting of TFA, TIPS, DCM and water.

Dialkyne Linker Synthesis

Dialkynyl linkers **13**-**15** contained a urea functionality and were readily synthesised from simple aliphatic alkynyl-amine building blocks in one step as depicted in **Scheme 4**. To obtain linker **15**, DIPEA was used to free-base the amine to give pentargyl amine **16**. The nucleophilic substitution of 1,1′-carbonyldiimidazole (CDI) **17** with alkynyl-amines **16, 18** and **19** proceeded in mostly good yield.

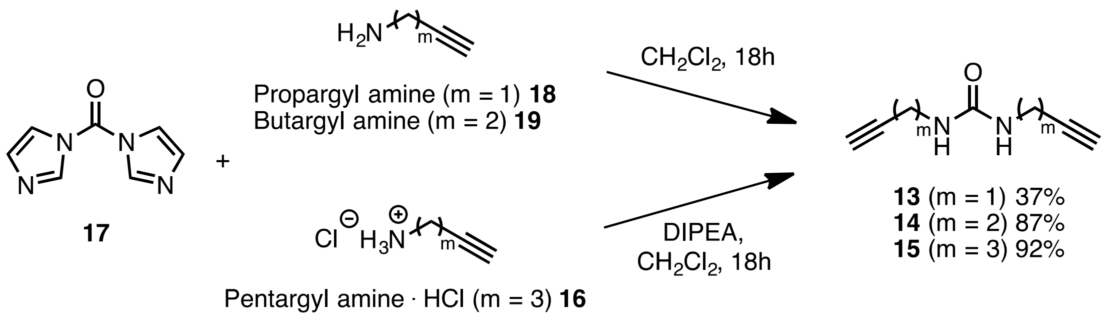

Scheme 4. Synthesis of urea-based linkers **13**-**15**

Manual Fmoc-Solid Phase Peptide Synthesis

Peptides were synthesised using Rink Amide MBHA resin (loading: 0.37 mmol/g) on a Vac-Man Laboratory Vacuum Manifold (Promega) using Fmoc-solid phase peptide synthesis (Fmoc-SPPS). Reactions were conducted in disposable 12 mL syringes with 20 µm frits (Agilent Technologies). Resin swelling was carried out with DCM (4 mL) for 5 min and the resin was then rinsed with DMF (3 × 4 mL).
Fmoc-protecting groups were removed by stirring with 20 % piperidine in DMF (3 × 4 mL) for 2 min, 20 min and 20 min followed by DMF washes (4 × 4 mL). Fmoc-protected amino acids were pre-activated with HATU (4 eq) and DIPEA (8 eq) in DMF (1 mL) on the shaker for 2 min followed by addition to the resin. Amino acid coupling reactions were sealed and shaken for 1 h. Double-couplings (2 × 30 min) were conducted with Arg. Fmoc-protected amino acids used included 6-Ahx, Arg (Pbf), Asp (Trt), Lys (Boc), Met, Phe and Thr (^{t}Bu).

The Chloranil test was used to check for amino acid coupling completion. Beads were rinsed with DCM (3 × 4 mL). In a separate vial, a saturated solution of Chloranil in toluene (50 µL) and acetaldehyde (200 µL) was added to a small proportion of the beads and incubated on a shaker for 5 min. A bead colour of either yellow/orange indicated complete coupling whereas green/brown/black indicated

incomplete coupling. For incomplete reactions the beads were subjected to further coupling conditions. The beads were washed with DMF (3 × 4 mL) to continue.

N-terminal capping with TAMRA-5 (12 eq) was accomplished by pre-activating the dye with HATU (6 eq) and DIPEA (6 eq) in DMF (1 mL) on the shaker for 2 min and then shaking for 3 h. The resin was washed with DMF (3 × 4 mL) and DCM (3 × 4 mL) before peptide cleavage and deprotection.

Peptides were cleaved off the resin and all side-chain protecting groups were removed by shaking with a 5 mL solution of TFA (87.5 %), H_2O (5 %), DCM (5 %) and triisopropylsilane (TIPS) (2.5 %) for 2 h. The cleaved mixture was filtered through cotton wool and washed with TFA (3 × 500 µL). Solutions containing the cleaved peptide were then blown down with nitrogen and triturated with Et_2O (2 × 2 mL) before LCMS analysis and semi-preparative HPLC purification.

Automated Fmoc-Solid Phase Peptide Synthesis

Automated peptide synthesis was carried out on a Liberty Blue Automated Microwave Peptide Synthesiser (CEM) using Rink Amide MBHA resin (loading: 0.37 mmol/g). All peptides were synthesised using Fmoc-protected amino acids (5 eq) and HATU (5 eq) in DMF, and DIPEA in NMP (2 M, 10 eq). Amino acids were coupled for 15 min with microwave irradiation using 25 W power at 75 °C. Arginine was double-coupled for 15 min each and without microwave irradiation. Fmoc-deprotection was achieved by microwave heating with 25 W power at 75 °C with 20 % piperidine in DMF for 3 min. Addition of the 6-Ahx linker amino acid, TAMRA-5 capping and the final deprotection and cleavage were conducted manually.

Constrained Peptides Synthesis by Copper Double-Click Reaction

All synthesised peptides were constrained with the synthesised linkers using copper-click chemistry resulting in the formation of two 1,2,3-triazole rings. Peptides containing two unnatural azido amino acids were doubly stapled or constrained with dialkynyl linkers using copper click chemistry under an inert nitrogen atmosphere. A solution of 1:1 $^tBuOH:H_2O$ (1 mL/mg peptide) containing the peptide (1 eq) and the linker (1.1 eq) were degassed with nitrogen for 15 min. A solution of degassed water (0.1 mL/mg peptide) containing $CuSO_4 \cdot 5H_2O$ (1 eq), THPTA (1 eq) and sodium ascorbate (3 eq) was transferred to the reaction mixture. The reaction was stirred for 16 h, lyophilised and purified by semi-automated HPLC purification.

Direct Fluorescence Polarisation Assay

Dissociation constants (K_d) for the interaction of TAMRA-5-labelled stapled peptides with mImportin α1 ΔIBB were determined by direct fluorescence polarisation (FP) measurement. TAMRA-5 belongs to the family of rhodamine dyes and has been used extensively for the study of PPIs [31] and DNA-protein interactions [26] by FP assays.[31-32] The mImportin α1 ΔIBB isoform was used since it is well characterised and has been used previously.[33] A one-site binding model was assumed with mImportin α1. mImportin α1 ΔIBB protein was purified in FP buffer (20 mM HEPES (pH 8), 200 mM NaCl) and 1 mM stock solutions of peptides were made up in FP buffer with 3 % v/v DMSO. Accurate mImportin α1 ΔIBB and stapled peptide concentrations were determined by amino acid analysis (PNAC facilities, Department of Biochemistry, University of Cambridge). 15-point dose-response curves were constructed using 2.0-fold serial dilutions of mImportin α1 ΔIBB in a mixture with a final concentration of 60 nM TAMRA-labelled peptide. 20 µL reaction volumes were pipetted into 96-well black polystyrene assay plates (Corning 384 Well Low Flange Black Flat Bottom Polystyrene NBS Microplate) followed by a 15 min incubation at room temperature with plates sealed with sealing tape (Corning 384 Well Microplate Aluminum Sealing Tape). Using an excitation wavelength of 540 nm and an emission wavelength of 590 nm, fluorescence polarisation was read at room temperature with a PHERAstar FS (BMG Labtech). All FP binding experiments were conducted in triplicates and a TAMRA-5 labelled stapled peptide only was used as a negative control. Assuming a one-site binding model of the stapled peptide with mImportin α1 ΔIBB the MARS Software Package (BMG Labtech) was used to fit a curve using a 4-parameter fitting model.

In order to determine the optimal incubation time for of HNF1β NLS tracer (Pep0: TAMRA-Ahx-TNKKMRRNRFK-NH_2) binding to take place before measuring anisotropy, a time-course experiment was conducted first (**Figure 5**). It was established that a 15 min incubation time was sufficient before measuring the fluorescence polarisation due to fast binding kinetics.

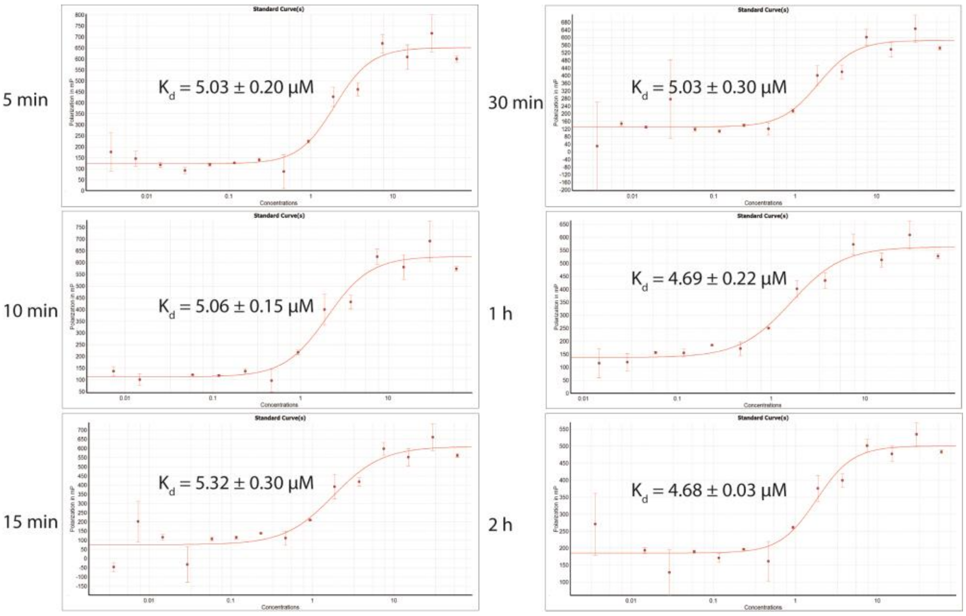

Figure 5. Time-course experiment to assess binding kinetics of HNF1β NLS tracer (Pep0: TAMRA-Ahx-TNKKMRRNRFK-NH_2) with mImportin α1 ΔIBB

TAMRA-5 alone bound to mImportin α1 with a K_d of approximately 122 μM (**Figure 6**).

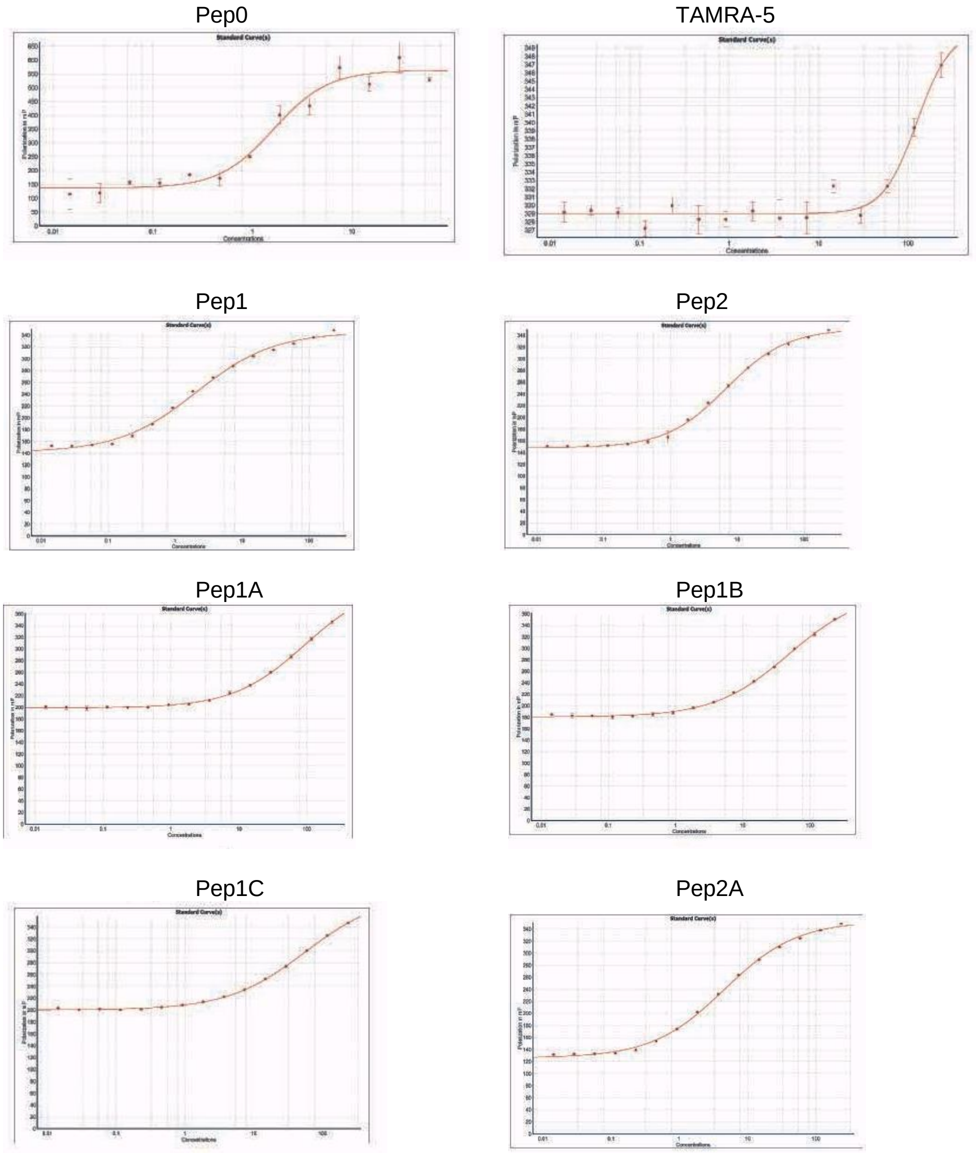

Figure 6. Direct fluorescence polarization curves. Red error bars show the standard deviation error of triplicate experiments.

General Chemistry Experimental

All non-aqueous reactions were carried out in oven-dried glassware under an atmosphere of dry nitrogen. Petroleum ether refers to the fraction boiling at 40–60 ◦C.

Analytical thin layer chromatography (TLC) was carried out on cut silica gel plates (Merck) and visualised by ultraviolet irradiation (λmax = 254 or 365 nm), potassium permanganate stain or vanillin stain. Flash column chromatography was carried out using silica gel (Merck, 230–400 mesh) under either compressed air or dry nitrogen and the solvent system was recorded in parentheses.

Semi-preparative high performance liquid chromatography (HPLC) was carried out on an Agilent 1260 Infinity System using a Supelcosil ABZ+PLUS column (250 mm × 21.2 mm, 5 μm). Samples were eluted with a linear gradient system (solvent A: 0.1 % (v/v) TFA in water, solvent B: 0.05 % (v/v) TFA in MeCN) over 20 min with a flow rate of 20 mL/min. Elution was monitored by UV absorbance at 220 and 254 nm. TAMRA-labelled peptides were also monitored for absorbance at 550 nm.

Liquid-chromatography mass spectra (LCMS) were recorded on an Acquity SQD UPLC/MS system (Waters) using a Supelcosil ABZ+PLUS column (33 mm × 4.6 × 3 μm) using the MassLynx 4.1 software. The following conditions were used to elute the samples: Solvent A: 10 mM ammonium acetate and 0.1 % formic acid in water; Solvent B: 95 % acetonitrile, 5 % water and 0.05 % formic acid. Samples were eluted with a linear gradient of 0 % B (0.0-0.7 min), 0-100 % B (0.7-4.2 min), 100 % B (4.2-7.7 min) and 100-0 % B (7.7-8.5 min) with a flow rate of 1 mL/min and an injection volume of 10 μL.

High-resolution mass spectrometry (HRMS) was conducted by the Mass Spectrometry Service at the Department of Chemistry, University of Cambridge, using a Bruker Bioapex 4.7eFTICR or a Micromass LCT Premier spectrometer. Mass-to-charge ratios (*m/z*) were reported in Daltons with their percentage abundance

and the relative fragment ions in parentheses. High-resolution values were calculated to four decimal places from the molecular formula, all found values were within a tolerance of 5 ppm.

^{1}H and ^{13}C NMR spectra were recorded at 298 K on a Bruker DPX-400 (400 MHz) and on a Bruker Avance 500 Cryo Ultrashield (500 MHz) spectrometer. Chemical shifts (δ_H, δ_C) were quoted in parts per million (ppm) relative to tetramethylsilane (δ = 0 ppm) and were referenced to the residual solvent signal. $CDCl_3$ or d_6-DMSO were used as internal deuterium locks. Assignments were made on the basis of chemical shifts, coupling constants and were supported by COSY, HSQC and DEPT 135 spectra if required. ^{1}H NMR chemical shifts were quoted to the nearest 0.01 ppm and ^{13}C NMR chemical shift to the nearest 0.1 ppm. For convenience the following abbreviations were used: app – apparent, br – broad, s – singlet, d – doublet, t – triplet, q – quartet, quint – quintet, m – multiplet, dd – doublet of doublets and so on. Quaternary carbons were denoted as C_Q. Coupling constants (*J*) were given in Hertz (Hz) and were rounded to the nearest 0.5 Hz.

Infra-Red (IR) spectra were recorded on a Perkin Elmer Spectre one FT-IT spectrophotometer and absorption maxima are reported in wavenumbers (cm^{-1}).

Synthesis of Azido Amino Acids

Fmoc-Aza-OH (1)

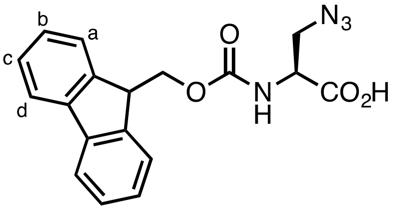

Prepared according to the procedure of Lau *et al.*[21] To a stirred solution of [Bis(trifluoroacetoxy)iodo]benzene (6.01 g, 14.0 mmol) in 2:1 DMF:H_2O (66 mL) was added Fmoc-Asn-OH **3** (3.30 g, 9.31 mmol). After 15 min, pyridine (1.51 mL, 18.6 mmol) was added and the reaction was stirred at room temperature for 14 h. The solvent was removed under reduced pressure and the oily residue was dissolved in water (50 mL). Concentrated aqueous HCl (1 mL) was added and the acidified solution was washed with Et_2O (4 × 30 mL). The aqueous phase was adjusted to pH 6 with 2 M NaOH solution. The resulting white precipitate was filtered, washed with water (5 × 30 mL), ice-cold EtOH (10 mL), Et_2O (10 × 10 mL) and dried *in vacuo* to give crude Fmoc-Dap-OH **5** (1.50 g, 4.58 mmol) as a beige powder. The compound was reacted on without further purification. **HRMS** (ES^+): Calcd for $[M+H]^+$ $C_{18}H_{19}O_4N_2$: 327.1339; found: 327.1332. Crude Fmoc-Dap-OH **5** (1.50 g, 4.58 mmol) was dissolved in a biphasic mixture of water (25 mL), MeOH (50 mL) and CH_2Cl_2 (50 mL). $CuSO_4 \cdot 5H_2O$ (6.87 mg, 0.028 mmol) and imidazole-1-sulfonyl azide hydrogen sulphate **7** (2.88 g, 13.8 mmol) were added and the mixture was adjusted to pH 9 with saturated aqueous K_2CO_3 solution. After stirring vigorously for 18 h, the reaction mixture was diluted with CH_2Cl_2 (50 mL) and the aqueous phase was isolated. The organic phase was extracted with saturated aqueous K_2CO_3 solution (2 × 80 mL). The combined aqueous extracts were washed with Et_2O (2 × 80 mL), dried ($MgSO_4$) and concentrated *in vacuo* to give Fmoc-Aza-OH **1** (1.57 g, 4.44 mmol, 48%) as a white solid. **m.p.** 123-125 °C (lit. m.p. [34] 119-120 °C); **IR** (ATR): 3285 (br, C-H), 2105 (azide), 1688 (C=O), 1257 (C-O), 1230 (C-N) cm^{-1}; ***m/z*** (ES^+) 327.2 ($[M+H]^+$, 100%); **^{1}H NMR** (500 MHz, $CDCl_3$) 7.77 (2H, d, *J* = 7.5, 2 × Ar**H$_d$**), 7.63-7.56 (2H, m, 2 × Ar**H$_a$**), 7.41 (2H, t, *J* = 7.5, 2 × Ar**H$_c$**), 7.32 (2H, t, *J* = 7.5, 2 × Ar**H$_b$**), 5.69 (1H, br s, N**H**), 4.62-4.54 (1H, m, α-C**H**), 4.49-4.39 (2H, m, C**H$_2$**O), 4.27-4.18 (1H, m, C**H**CH_2O), 3.87-3.74 (2H, m, β-C**H$_2$**); **^{13}C NMR** (125 MHz, $CDCl_3$) 173.7 (**C**O_2H), 156.0 (**C**(O)NH), 143.6 (2 × **Ar$_Q$**), 141.3 (2 × **Ar$_Q$**), 127.8 (2 × **Ar$_d$**), 127.3 (2 × **Ar$_a$**), 125.0 (2 × **Ar$_c$**), 120.0 (2 × **Ar$_b$**), 67.5 (**C**H_2O), 53.7 (α-**C**H_2), 52.2 (β-**C**H_2), 47.0 (**C**HCH_2O); ***m/z*** (ES^-) 351.1 ($[M-H]^-$, 100%); **HRMS** (ES^+): Calcd for $[M+Na]^+$ $C_{18}H_{16}O_4N_4{}^{23}Na_1$: 375.1064; found: 375.1054; **$[\alpha]_D^{25}$** -34.0 (c = 0.1, DMF), lit. [21] $[\alpha]_D^{27}$ -10.3 (c = 1.0, DMF).
The data is in accordance with the literature.[21]

Fmoc-Aha-OH (2)

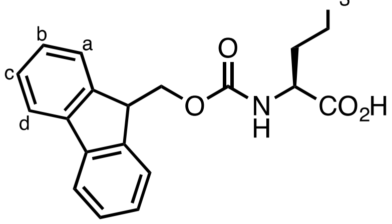

Prepared according to the procedure of Lau *et al.*[21] and in a similar manner to **1** from Fmoc-Gln-OH **4** (3.30 g, 8.96 mmol), [bis(trifluoroacetoxy)iodo]benzene (5.78 g, 13.4 mmol) and pyridine (1.45 mL, 17.9 mmol) to give crude Fmoc-Dab-OH **6** (1.63 g, 4.79 mmol) as a beige powder. The compound was reacted on without further purification. ***m/z*** (ES^+) 341.2 ($[M+H]^+$, 100%); **HRMS** (ES^+): Calcd for $[M+H]^+$ $C_{19}H_{21}O_4N_2$: 341.1496; found: 341.1499. Fmoc-Aha-OH **2** was prepared in a similar manner to **1** from Fmoc-Dab-OH **6** (1.63 g, 4.79 mmol), $CuSO_4 \cdot 5H_2O$ (5.74 mg, 0.023 mmol) and imidazole-1-sulfonyl azide hydrogen sulphate **7** (3.01 g, 14.4 mmol) to give Fmoc-Aha-OH **2** (1.14 g, 3.12 mmol, 35%) as white crystals. **m.p.** 122-

124 °C (lit. m.p. [35] 128 °C); **IR** (ATR): 3322 (br, C-H), 2105 (azide), 1693 (C=O), 1530, 1252 (C-O), 1087, 1046 cm^{-1}; ***m/z*** (ES^+) 327.2 ($[M+H]^+$, 100%); **^{1}H NMR** (400 MHz, $CDCl_3$) 7.74 (2H, d, *J* = 7.5, 2 × Ar**H$_d$**), 7.60-7.58 (2H, m, 2 × Ar**H$_a$**), 7.40-7.37 (2H, m, 2 × Ar**H$_c$**). 7.20 (2H, t, *J* = 7.0, 2 × Ar**H$_b$**), 4.53-4.27 (2H, m, CHC**H$_2$**O), 4.11-4.20 (1H, m, α-C**H**), 3.48-3.28 (3H, m, C**H**CH_2O, γ-C**H$_2$**), 2.21-2.03 (1H, m, $β_1$-C**H$_2$**), 1.98-1.93 (1H, m, $β_2$-C**H$_2$**); **^{13}C NMR** (100 MHz, $CDCl_3$) 178.1 (**C**O_2H), 157.9 (O**C**(O)N), 144.8 (2 × **Ar$_Q$**), 142.3 (2 × **Ar$_Q$**), 128.8 (2 × **Ar$_d$**), 128.1 (2 × **Ar$_a$**), 126.0 (2 × **Ar$_c$**), 121.0 (2 × **Ar$_b$**), 68.2 (**C**H_2O), 54.6 (α-**C**H), 48.9 (γ-**C**H_2), 48.0 (**C**HCH_2O), 32.2 (β-**C**H_2); ***m/z*** (ES^+) 367.1 ($[M+H]^+$, 100%); **HRMS** (ES^+): Calcd for $[M+H]^+$ $C_{19}H_{19}O_4N_4$: 367.1401; found: 367.1408; **$[α]_D^{25}$** -10.0 (c = 0.1, MeOH), lit. [21] $[α]_D^{27}$ -14.4 (c = 1.0, MeOH).
The data is in accordance with the literature.[21]

Imidazole-1-sulfonyl azide hydrogen sulfate (7)

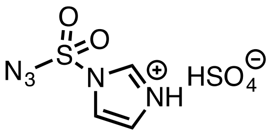

Prepared according to the procedure of Lau *et al*.[21] Sulfuryl chloride (16.1 mL, 200 mmol) was added dropwise over 30 min to an ice-cooled suspension of sodium azide (13.0 g, 200 mmol) in MeCN (200 mL). The resulting mixture was stirred at room temperature for 16 h. Imidazole (25.9 g, 380 mmol) was added to the ice-cooled mixture and the resulting slurry was stirred for 5 h at room temperature. The mixture was diluted with EtOAc (400 mL) and water (400 mL). The organic layer was washed with water (400 mL), saturated aqueous $NaHCO_3$ (2 × 400 mL) and dried over $MgSO_4$ and filtered before the volume was reduced to approximately 200 mL by concentrating *in vacuo*. Concentrated sulfuric acid (11 mL) in EtOAc (100 mL) was added dropwise over 30 min at 0 °C. The reaction mixture was warmed to room temperature and stirred for 16 h. The precipitate was filtered, washed with EtOAc (3 × 60 mL) and dried *in vacuo* to give the title compound **7** (43.8, 161.3 mmol, 81%) as a white, crystalline powder. All aqueous phases were quenched with sodium nitrite and acid before disposal. * refers to the NMR shifts of a decomposition product. **IR** (ATR): 2177 (azide), 1429, 1301 (S=O), 1232, 1155, 1098 cm^{-1}; ***m/z*** (ES^+) 327.2 ($[M+H]^+$, 100%); **^{1}H NMR** (500 MHz, d_6-DMSO) 14.14 (1H, br s, N**H**$^+$), 13.89 (1H, br s, **H**$SO4^-$), 9.20* (1H, t, *J* = 1.5, NC**H**N), 8.99-8.96 (1H, m, NC**H**), 7.73 (1H, t, *J* = 1.5, NC**H**), 7.60* (2H, d, *J* = 1.5, NHC**H**), 7.59-7.57 (1H, m, NC**H**N); **^{13}C NMR** (125 MHz, d_6-DMSO) 134.3 (N**C**HN), 134.0* (N**C**HN), 120.2 (N**C**HCH), 119.8 (HN**C**HCH), 119.3* (HN**C**HCH); ***m/z*** (ES^+) 174.0 ($[M+H]^+$, 100%); **HRMS** (ES^+): Calcd for $[M+H]^+$, $C_3H_4O_2N_5{}^{32}S_1$: 174.0080; found: 174.0076.
The data is in accordance with the literature.[22]

Synthesis of Dialkynyl Linkers

1,3-Di(prop-2-yn-1-yl)urea (13)

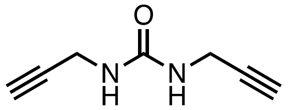

To a stirred solution of CDI **17** (300 mg, 1.85 mmol) in CH_2Cl_2 (5 mL) was added propargylamine **18** (237 µL, 3.70 mmol) and the reaction was stirred for 18 h. The solvent was removed *in vacuo* and the crude product was purified by flash column chromatography (1-5% MeOH in CH_2Cl_2, R_f = 0.47 (5% MeOH in CH_2Cl_2)) to give **13** (94.0 mg, 0.690 mmol, 37%) as white crystals. **m.p.** 192-194 °C (lit. m.p. [36] 187 °C); **IR** (ATR): 3315 (C-H), 1594 (C=O), 1426, 1355, 1288, 1251 cm^{-1}; ***m/z*** (ES^+) 327.2 ($[M+H]^+$, 100%); **^{1}H NMR** (400 MHz, d_6-DMSO) 6.31 (2H, t, *J* = 6.0, 2 × N**H**), 3.78 (4H, dd, *J* = 6.0, 2.5, 2 × C**H$_2$**), 3.04 (2H, t, *J* = 2.5, 2 × CC**H**); **^{13}C NMR** (100 MHz, d_6-DMSO) 156.9 (C=O), 82.3 (2 × **C$_Q$**), 72.6 (2 × C**C**H), 28.8 (2 × **C**H_2); ***m/z*** (ES^+) 137.1 ($[M+H]^+$, 100%); **HRMS** (ES^+): Calcd for $[M+H]^+$, $C_7H_9O_1N_2$: 137.0709; found: 137.0707.
The data is in accordance with the literature.[37]

1,3-Di(but-3-yn-1-yl)urea (14)

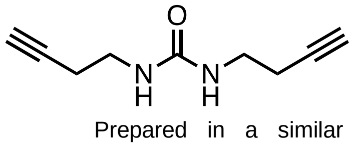

Prepared in a similar manner to **13** from 1-amino-3-butyne **19** (500 mg, 7.23 mmol) and CDI **17** (587 mg, 3.62 mmol) to give **14** (519 mg, 3.16 mmol, 87%) as white crystals. **m.p.** 167-169 °C decomp. (lit. m.p. [38] 146 °C); **IR** (ATR): 3322 (C-H), 1594 (C=O), 1448, 1365, 1262, 1047 cm^{-1}; ***m/z*** (ES^+) 327.2 ($[M+H]^+$, 100%); **^{1}H NMR** (400 MHz, d_6-DMSO) 6.12 (2H, t, *J* = 6.5, 2 × N**H**), 3.12-3.06 (4H, m, 2 × NHC**H$_2$**), 2.81 (2H, t, *J* = 2.5, 2 × CC**H**), 2.23 (4H, td, *J* = 6.5, 2.5, 2.5, 2 × HCCC**H$_2$**); **^{13}C NMR** (100 MHz, d_6-DMSO) 157.7 (**C**=O), 82.8 (2 × **C$_Q$**), 72.1 (2 × C**C**H), 38.6 (2 × N**C**H_2), 19.8 (2 × NCH_2**C**H_2); ***m/z*** (ES^+) 165.1 ($[M+H]^+$, 100%); **HRMS** (ES^+): Calcd for $[M+H]^+$, $C_9H_{13}O_1N_2$: 165.1022; found: 165.1019.

1,3-Di(pent-4-yn-1-yl)urea (15)

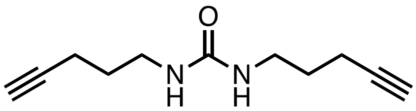

To a stirred solution of CDI **17** (339 mg, 2.09 mmol) in CH_2Cl_2 (5 mL) was added 4-pentyn-1-amine· HCl **16** (500 mg, 4.18 mmol) and DIPEA (801 µL, 4.60 mmol). The reaction was stirred for 18 h. The solvent was removed *in vacuo* and the crude product was purified by column chromatography to give **15** (368 mg, 1.92 mmol, 92%) as white crystals. **m.p.** 94-96 °C; **IR** (ATR): 3322 (C-H), 1580 (C=O), 1272, 1225 cm^{-1}; ***m/z*** (ES^+) 327.2 ($[M+H]^+$, 100%); **^{1}H NMR** (400 MHz, d_6-DMSO) 5.84 (2H, t, *J* = 5.5, 2 × N**H**), 3.06-2.99 (4H, m, 2 × C**H$_2$**NH), 2.76 (2H, t, *J* = 2.5, 2 × CC**H**), 2.13 (4H, td, *J* = 7.0, 2.5, 2 × HCCC**H$_2$**), 1.52 (4H, quint, *J* = 7.0, 2 × CH_2C**H$_2$**CH_2); **^{13}C NMR** (100 MHz, d_6-DMSO) 158.5 (**C**=O), 84.6 (2 × **C$_Q$**), 71.8 (2 × C**C**H), 38.8 (2 × N**C**H$_2$), 29.4 (2 × **C**H$_2$CCH), 15.7 (2 × CH_2**C**H$_2$$CH_2$); ***m/z*** ($ES^+$) 193.1 ($[M+H]^+$, 100%); **HRMS** ($ES^+$): Calcd for $[M+H]^+$, $C_{11}H_{17}O_1N_2$: 193.1341; found: 193.1342.

Synthesis of TAMRA-5

4-(4-(Dimethylamino)-2-hydroxybenzoyl)isophthalic acid (11)

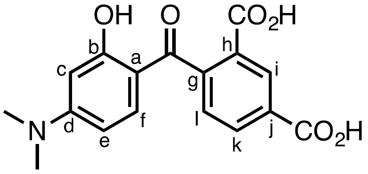

Trimellitic anhydride **10** (10.0 g, 52.0 mmol) was added to a stirred solution of 3-dimethylamino phenol **9** (5.92 g, 43.2 mmol) in toluene (140 mL) at 60 °C. The reaction was heated at reflux overnight in the absence of light. Upon cooling to room temperature, the resulting purple precipitate was filtered and washed with toluene (3 × 30 mL). The filtered solid was then heated at reflux in MeOH (160 mL) for 10 min, then glacial AcOH (40 mL) was added. After a further 10 min heating at reflux, the reaction was cooled and the solvent removed *in vacuo*, leaving a purple solid. Multiple washes with MeOH (3 × 50 mL) gave pure **11** (2.57 g, 7.80 mmol, 15%) as a grey solid. **m.p.** 250 °C decomp. (lit. m.p. [39] 265 °C); **IR** (ATR): 2912 (br, OH), 2583 (br, OH), 1691 (C=O), 1632 (C=O), 1557, 1350, 1209 cm^{-1}; ***m/z*** (ES^+) 327.2 ($[M+H]^+$, 100%); **^{1}H NMR** (500 MHz, d_6-DMSO) 13.42 (2H, br s, 2 × CO_2**H**), 12.37 (1H, s, O**H**), 8.49 (1H, d, *J* = 1.5, Ar**H$_i$**), 8.20 (1H, dd, *J* = 8.0, 1.5, Ar**H$_k$**), 7.52 (1H, d, *J* = 8.0, Ar**H$_l$**), 6.81 (1H, d, *J* = 9.0, Ar**H$_f$**), 6.20 (1H, dd, *J* = 9.0, 2.5, Ar**H$_e$**), 6.11 (1H, d, *J* = 2.5, Ar**H$_c$**), 3.00 (6H, s, 2 × N(C**H$_3$**)$_2$); **^{13}C NMR** (125 MHz, d_6-DMSO) 197.7 (**C**(O)), 166.2 (2 × **C**O$_2$H), 164.3 (**Ar$_b$**), 155.9 (**Ar$_d$**), 143.8 (**Ar**), 133.9 (**Ar**), 132.7 (**Ar**), 131.7 (**Ar**), 130.8 (**Ar**), 130.1 (**Ar**), 128.3 (**Ar**), 109.5 (**Ar**), 104.6 (**Ar**), 97.1 (**Ar$_c$**), 48.7 (N(**C**H$_3$)$_2$); ***m/z*** (ES^+) 330.2 ($[M+H]^+$, 100%); **HRMS** (ES^+): Calcd for $[M+H]^+$, $C_{17}H_{16}O_6N_1$: 330.0972; found: 330.0959.
The data is in accordance with the literature.[27]

5-Carboxytetramethylrhodamine (TAMRA-5) (8)

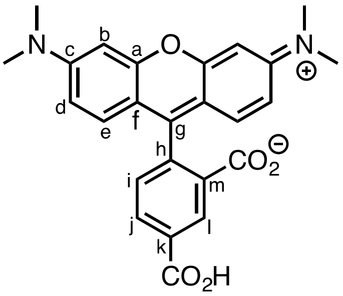

To a solution of 4-(4-(dimethylamino)-2-hydroxybenzoyl)isophthalic acid **11** (1.50 g, 4.56 mmol) and dimethylaminophenol **9** (814 mg, 5.94 mmol) in DMF (22 mL) was added trimethylsilyl polyphosphate (3.22 mL) and the solution was heated to 110 °C for 3 h in the absence of light. Upon cooling to room temperature the solvent was removed *in vacuo*. The crude residue was then dissolved in 5% aqueous NaOH solution (35 mL) and stirred for 18 h in the absence of light. The reaction mixture was then filtered and the precipitate was washed with water (70 mL). The combined filtrate was acidified with 6 M HCl until a precipitate formed. The precipitate was filtered, washed with water (50 mL), 1:10 EtOH : EtOAc (3 × 50 mL) and Et_2O (50 mL), then dried *in vacuo* to give TAMRA-5 **8** (1.96 g, 4.56 mmol, 51%) as a dark grey powder. **m.p.** 350 °C decomp; **IR** (ATR): 3395 (C-H), 2935 (br, OH), 1720 (C=O), 1591, 1343, 1185 cm^{-1}; ***m/z*** (ES^+) 327.2 ($[M+H]^+$, 100%); **^{1}H NMR** (400 MHz, d_6-DMSO) 8.54 (1H, s, Ar**H$_l$**), 8.31 (1H, d, *J* = 8.0, Ar**H$_j$**), 7.44 (1H, d, *J* = 8.0, Ar**H$_i$**), 6.76 (4H, br s, Ar**H$_b$**, Ar**H$_d$**), 6.69 (2H, br s, Ar**H$_e$**), 3.10 (12H, s, 4 × N(C**H$_3$**)$_2$); **^{13}C NMR** (100 MHz, d_6-DMSO) 167.0 (**C**(O)O), 166.0 (**C**(O)O), 163.0 (**Ar$_g$**), 154.1 (2 × **Ar$_a$**, 2 × **Ar$_c$**), 134.7 (**Ar$_k$**, **Ar$_m$**), 132.7 (**Ar$_e$**, **Ar$_j$**, **Ar$_l$**), 129.4 (**Ar$_i$**), 111.4 (2 × **Ar$_d$**), 108.8 (2 × **Ar$_f$**), 97.2 (2 × **Ar$_b$**), 34.0 (2 × N(**C**H$_3$)$_2$); ***m/z*** (ES^+) 431.2 ($[M+H]^+$, 100%); **HRMS** (ES^+): Calcd for $[M+H]^+$, $C_{25}H_{23}O_5N_2$: 431.1607; found: 431.1607.
The data is in accordance with the literature.[27]

^{1}H and ^{13}C NMR Spectra

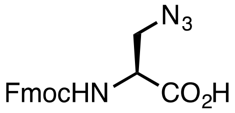

1

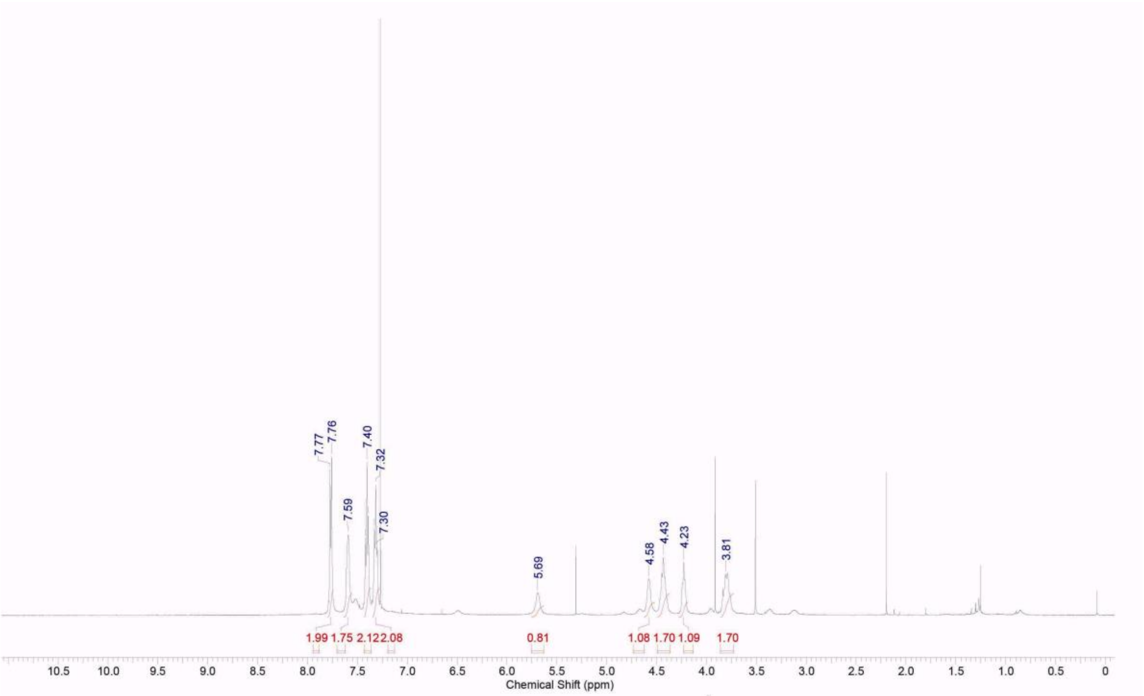

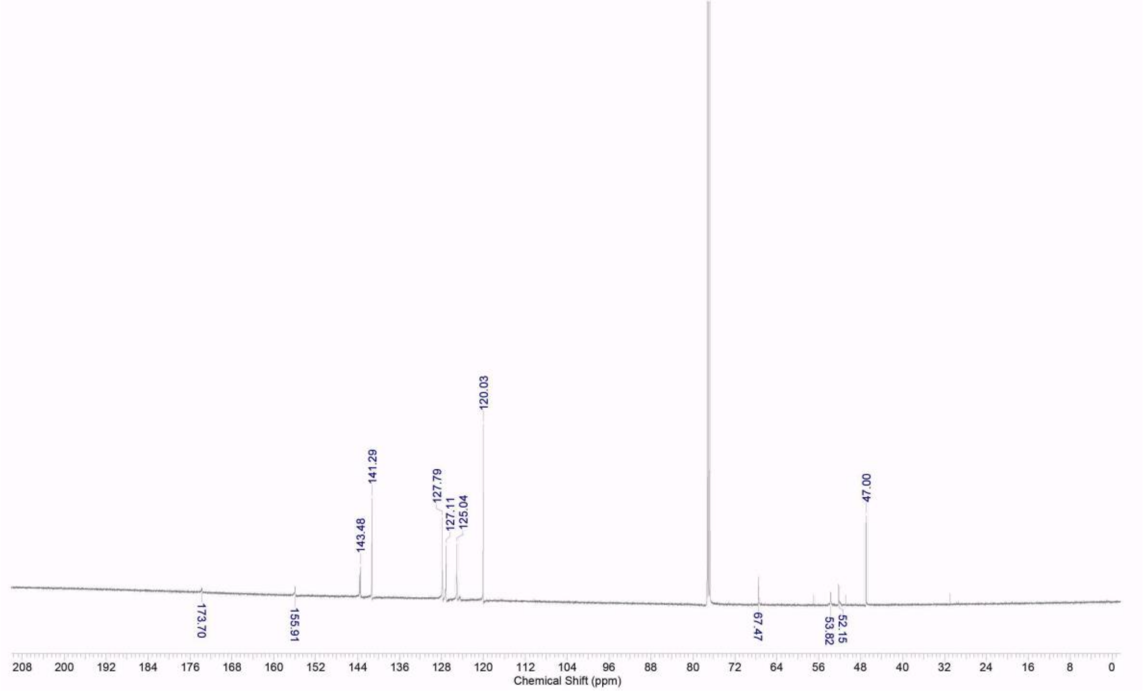

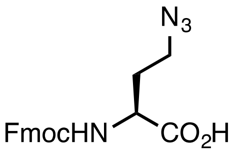
N3
FmocHN
CO2H

2

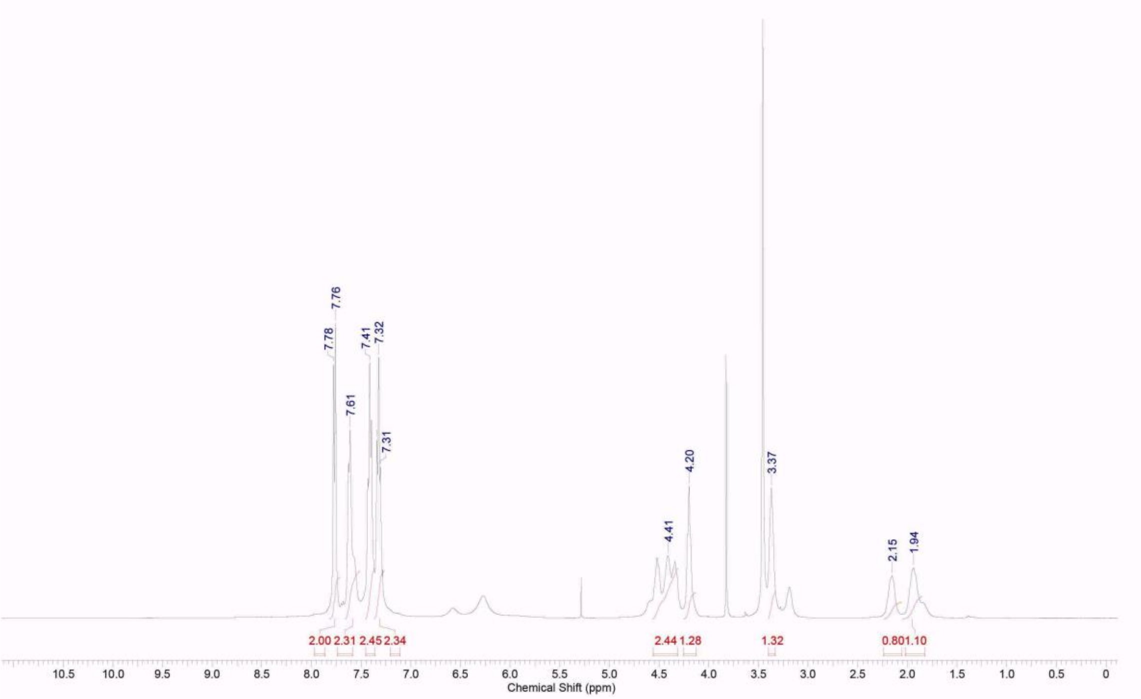
7.78
7.76
7.61
7.41
7.32
7.31
4.41
4.20
3.37
2.15
1.94
2.00 2.31 2.45 2.34
2.44 1.28
1.32
0.80 1.10
10.5 10.0 9.5 9.0 8.5 8.0 7.5 7.0 6.5 6.0 5.5 5.0 4.5 4.0 3.5 3.0 2.5 2.0 1.5 1.0 0.5 0
Chemical Shift (ppm)

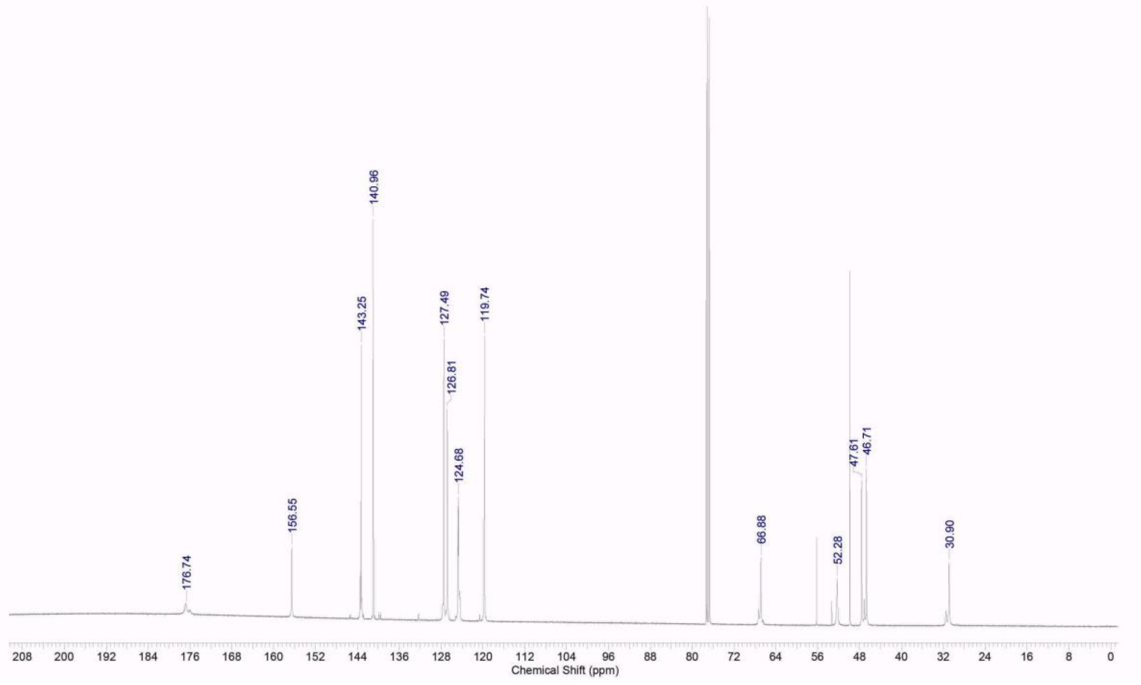
176.74
156.55
143.25
140.96
127.49
126.81
124.68
119.74
66.88
52.28
47.61
46.71
30.90
208 200 192 184 176 168 160 152 144 136 128 120 112 104 96 88 80 72 64 56 48 40 32 24 16 8 0
Chemical Shift (ppm)

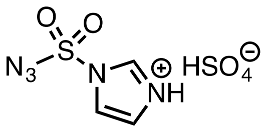

7

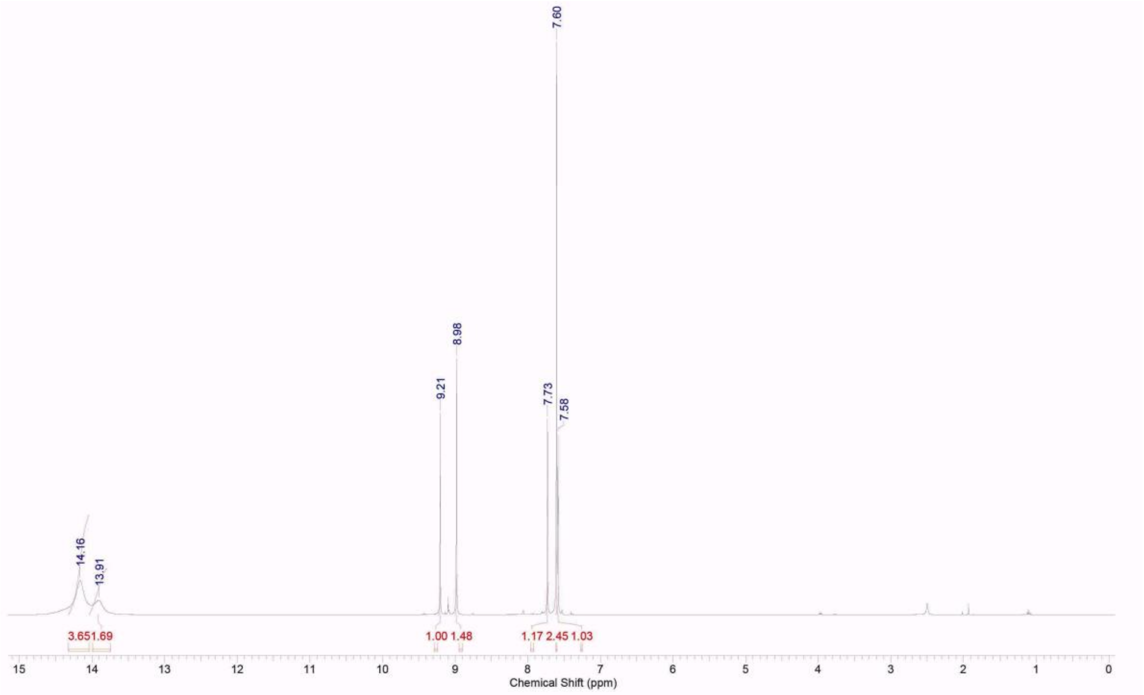
7.60
8.98
9.21
7.73
7.58
14.16
13.91
3.65 1.69
1.00 1.48
1.17 2.45 1.03
15 14 13 12 11 10 9 8 7 6 5 4 3 2 1 0
Chemical Shift (ppm)

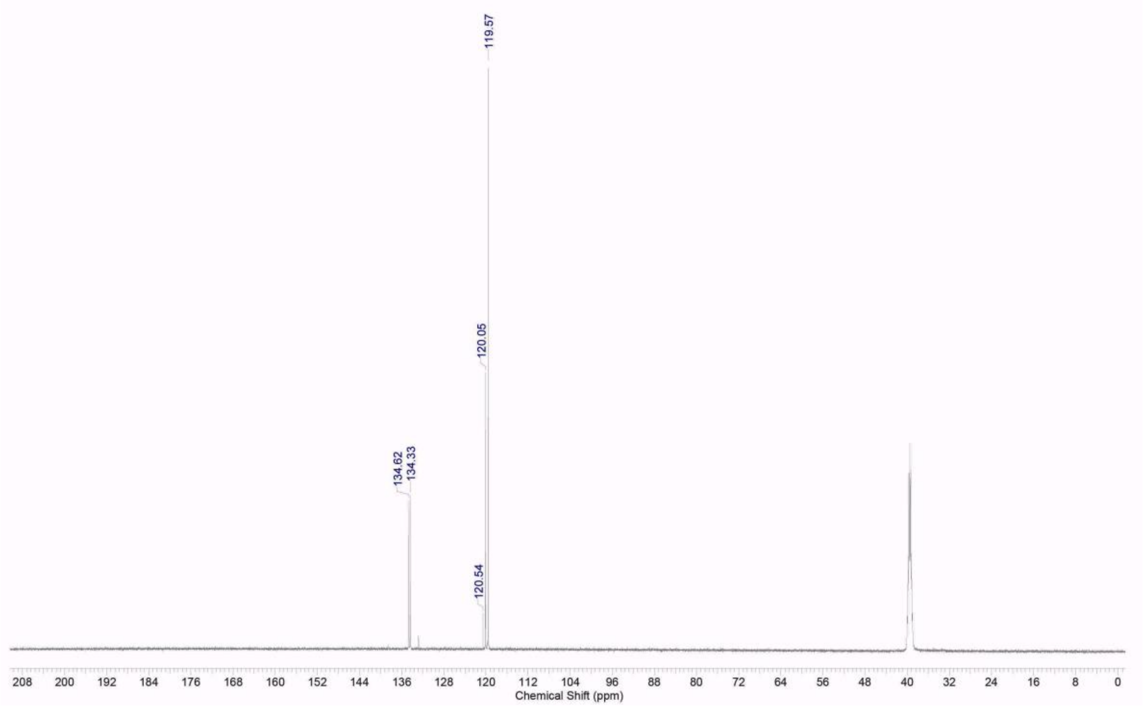
119.57
120.05
134.62
134.33
120.54
208 200 192 184 176 168 160 152 144 136 128 120 112 104 96 88 80 72 64 56 48 40 32 24 16 8 0
Chemical Shift (ppm)

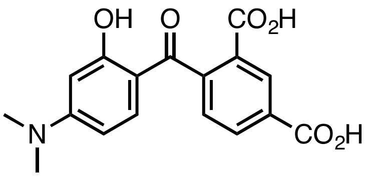

11

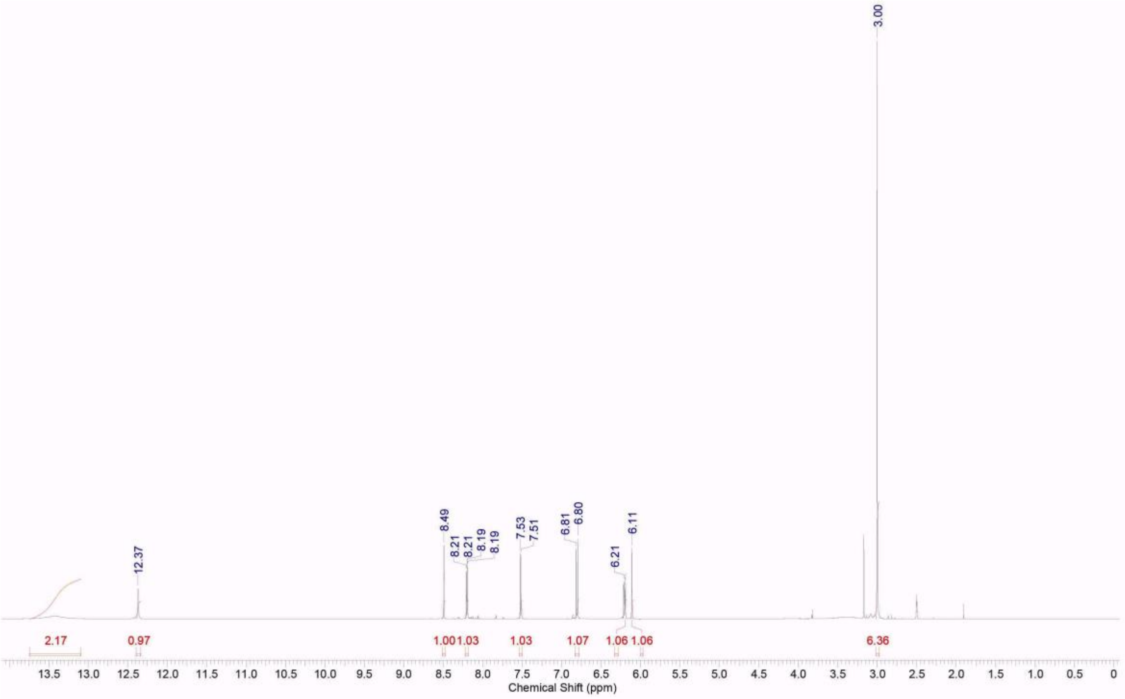

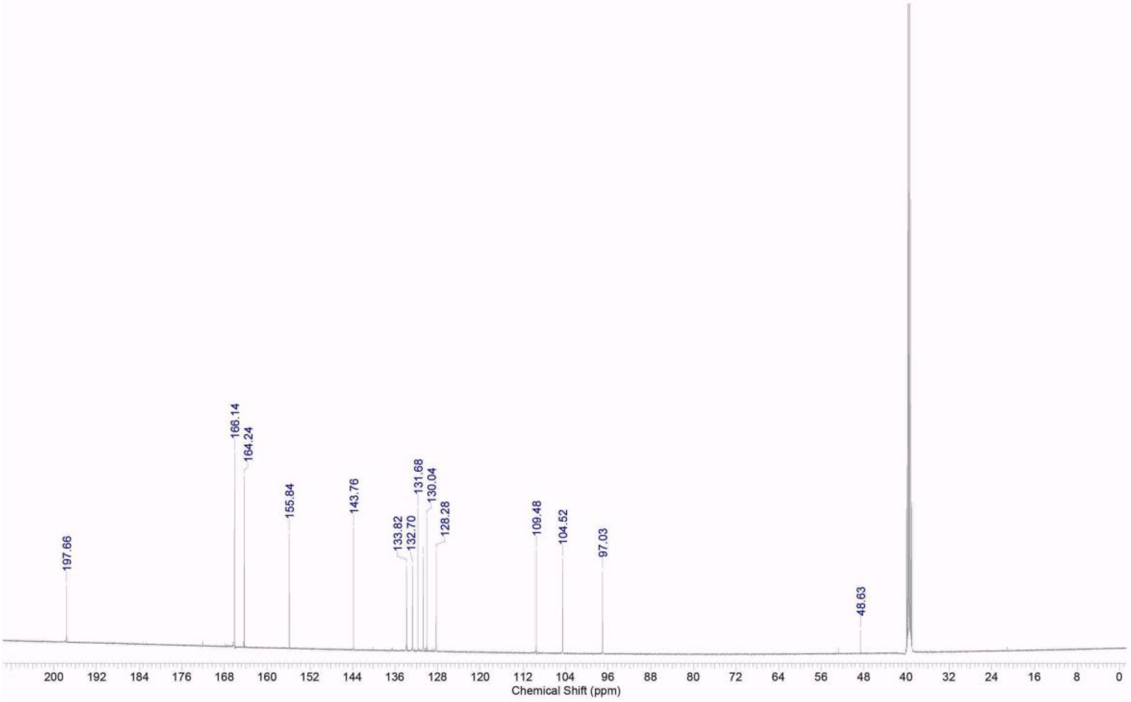

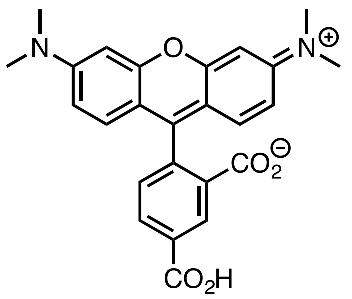

8

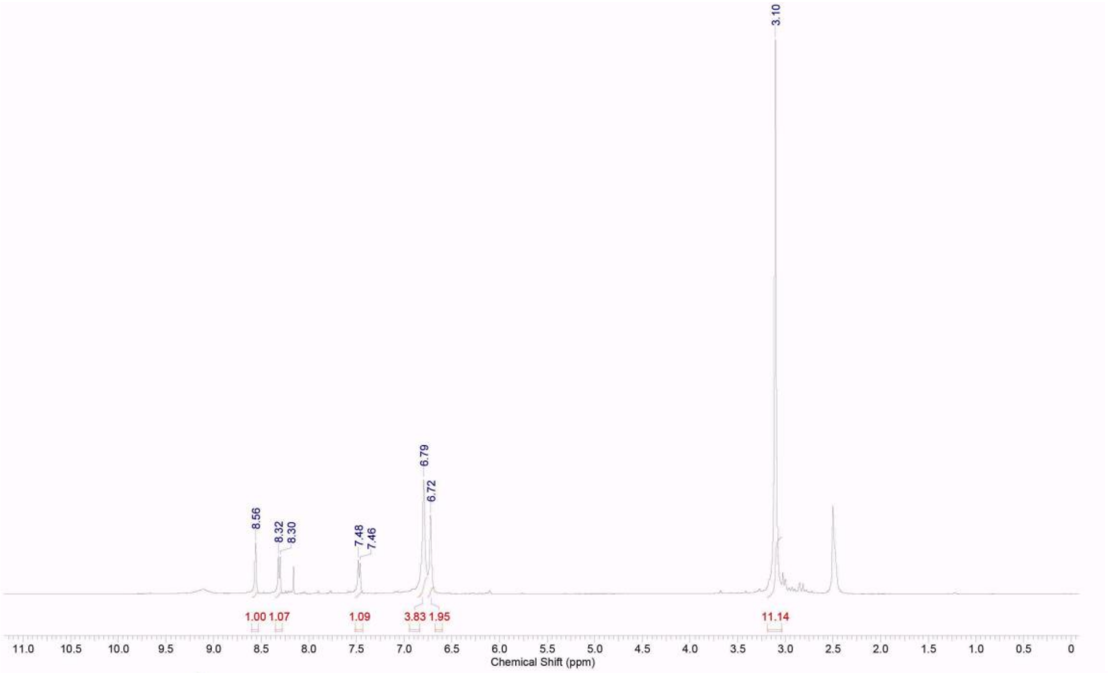

3.10
8.56
8.32
8.30
7.48
7.46
6.79
6.72
1.00 1.07
1.09
3.83 1.95
11.14
11.0 10.5 10.0 9.5 9.0 8.5 8.0 7.5 7.0 6.5 6.0 5.5 5.0 4.5 4.0 3.5 3.0 2.5 2.0 1.5 1.0 0.5 0
Chemical Shift (ppm)

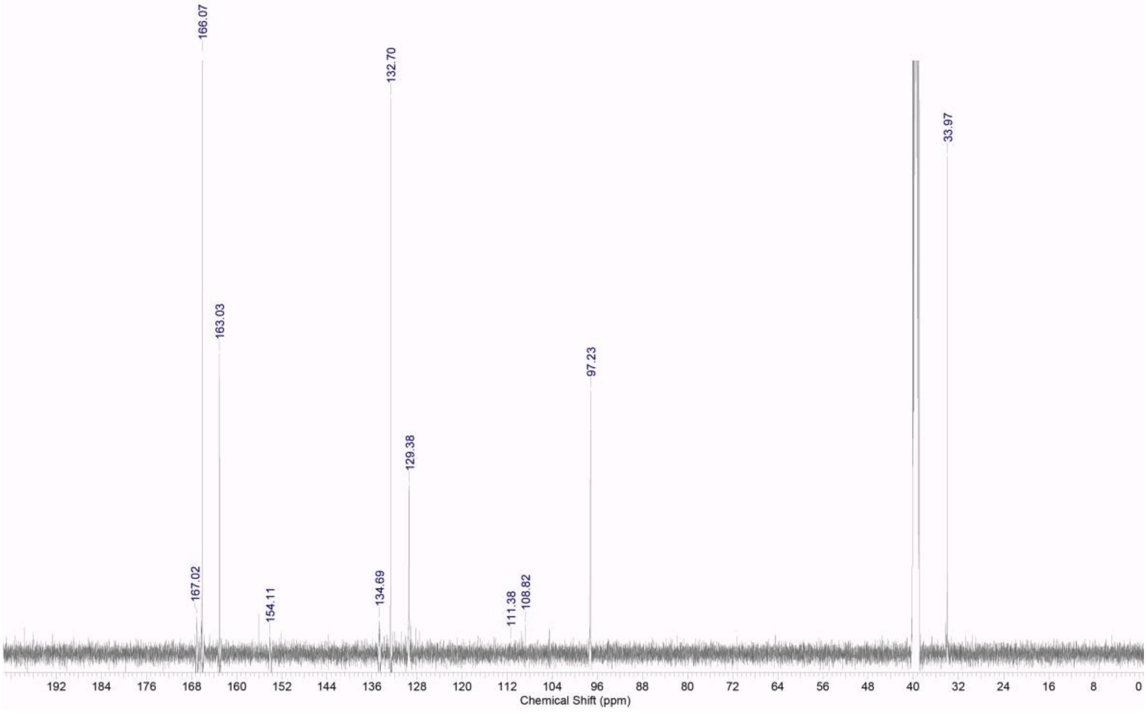

166.07
132.70
33.97
163.03
97.23
129.38
167.02
154.11
134.69
111.38
108.82
192 184 176 168 160 152 144 136 128 120 112 104 96 88 80 72 64 56 48 40 32 24 16 8 0
Chemical Shift (ppm)

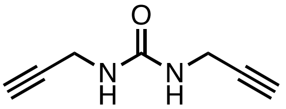
O
N
H
N
H

13

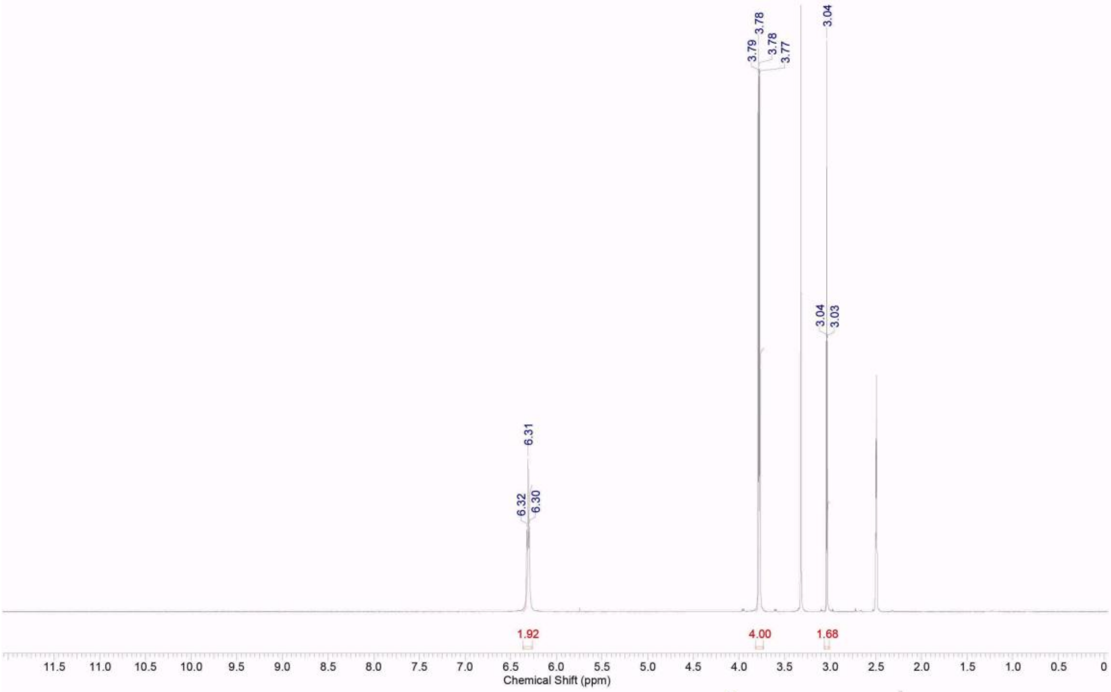
3.79
3.78
3.78
3.77
3.04
3.04
3.03
6.31
6.32
6.30
1.92
4.00
1.68
11.5 11.0 10.5 10.0 9.5 9.0 8.5 8.0 7.5 7.0 6.5 6.0 5.5 5.0 4.5 4.0 3.5 3.0 2.5 2.0 1.5 1.0 0.5 0
Chemical Shift (ppm)

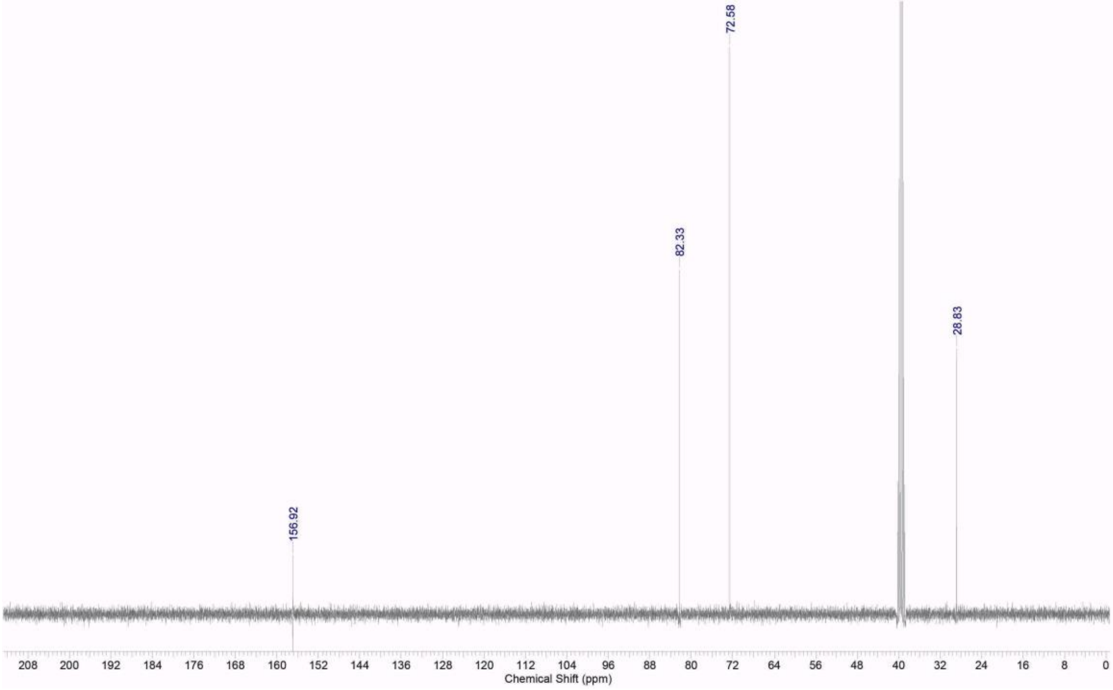
72.58
82.33
28.83
156.92
208 200 192 184 176 168 160 152 144 136 128 120 112 104 96 88 80 72 64 56 48 40 32 24 16 8 0
Chemical Shift (ppm)

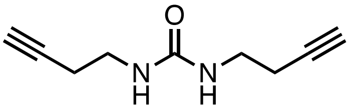
O
N
H
N
H

14

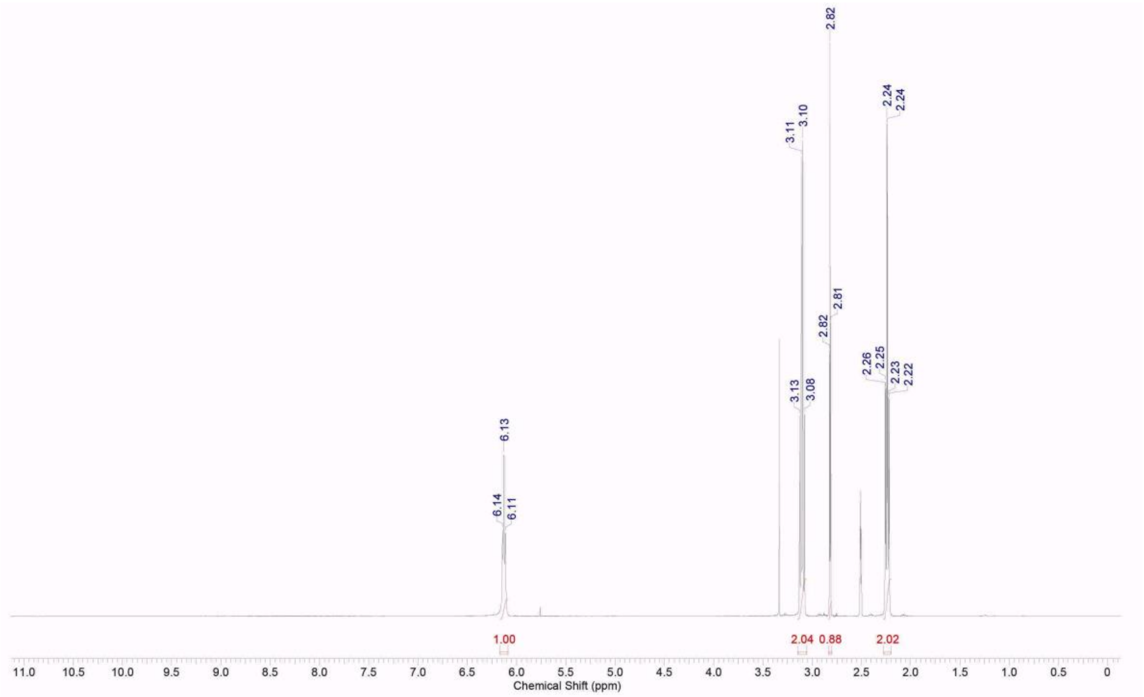
2.82
2.24
2.24
3.11
3.10
2.81
2.82
2.26
2.25
2.23
2.22
3.13
3.08
6.13
6.14
6.11
1.00
2.04
0.88
2.02
Chemical Shift (ppm)

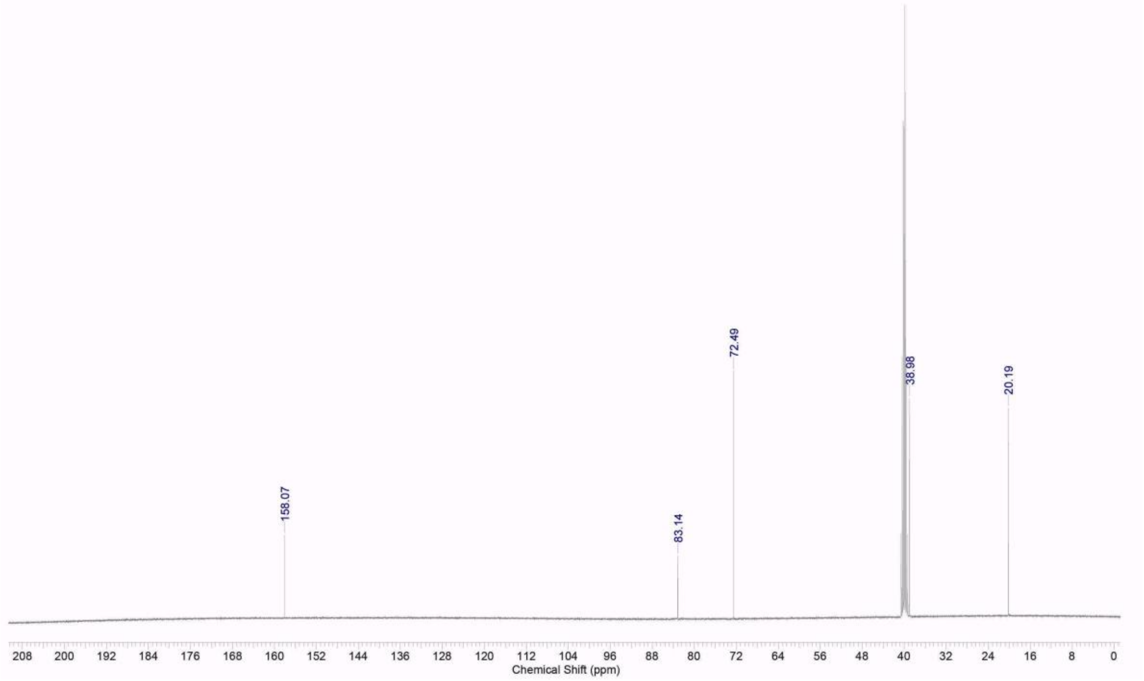
72.49
38.98
20.19
158.07
83.14
Chemical Shift (ppm)

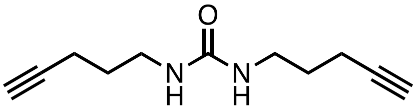
O
N
H
N
H

15

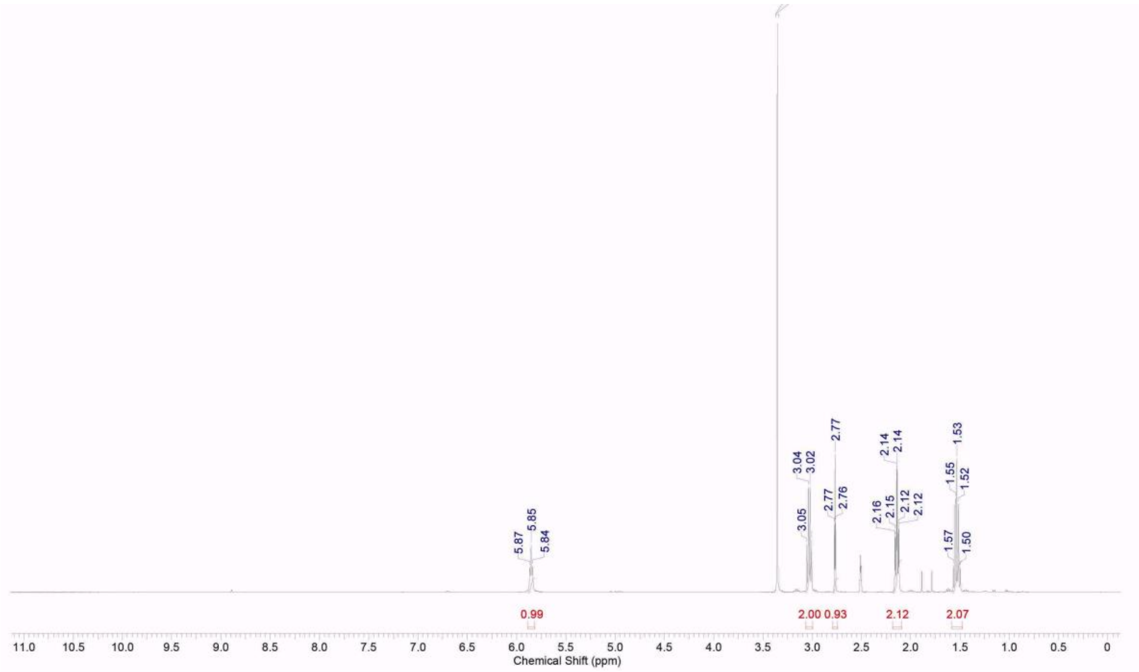
5.87
5.85
5.84
3.05
3.04
3.02
2.77
2.77
2.76
2.16
2.15
2.14
2.14
2.12
2.12
1.57
1.55
1.53
1.52
1.50
0.99
2.00
0.93
2.12
2.07
11.0 10.5 10.0 9.5 9.0 8.5 8.0 7.5 7.0 6.5 6.0 5.5 5.0 4.5 4.0 3.5 3.0 2.5 2.0 1.5 1.0 0.5 0
Chemical Shift (ppm)

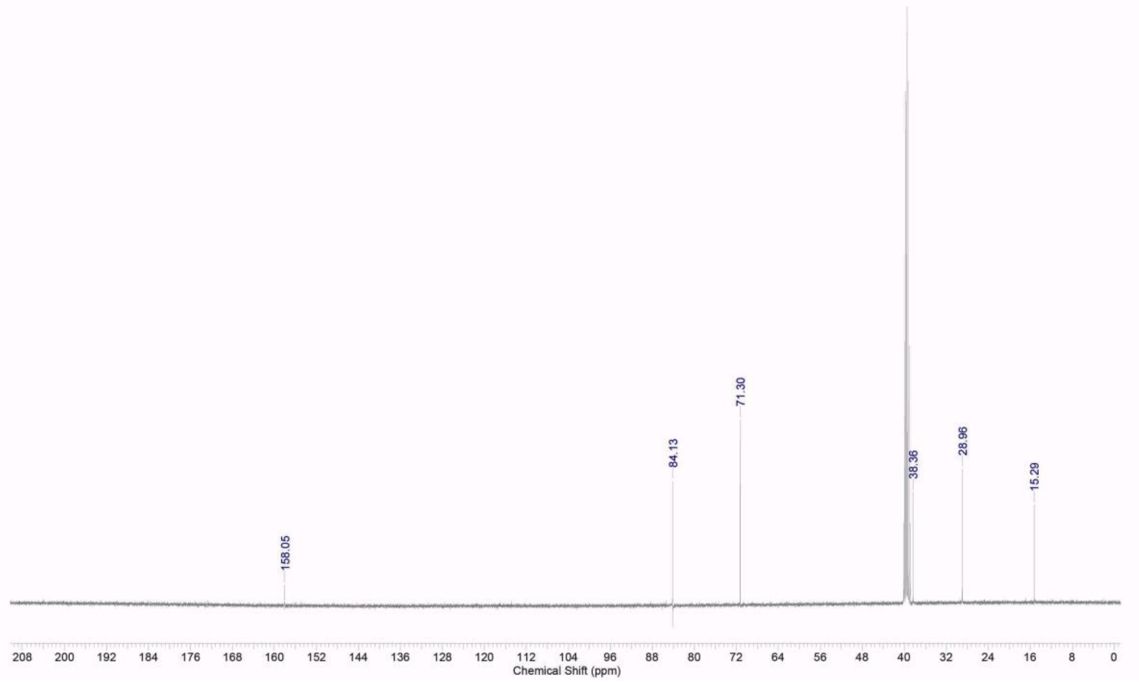
158.05
84.13
71.30
38.36
28.96
15.29
208 200 192 184 176 168 160 152 144 136 128 120 112 104 96 88 80 72 64 56 48 40 32 24 16 8 0
Chemical Shift (ppm)

Peptide Mass Spectrometry Characterisation Data

Peptide synthesis yields are summarised in Table 1.

Table 1 – Solid phase peptide synthesis yields

Peptide	Peptide Sequence	Percentage yield
**Pep0**	TAMRA-Ahx-TNKKMRRNRFK-NH_2	25
**Pep1**	TAMRA-Ahx-T-Aza-KKMRR-Aza-R-NH_2	5
**Pep2**	TAMRA-Ahx-T-Aha-KKMRR-Aha-R-NH_2	8

All peptides were purified by HPLC with a gradient of 20-60% over 20 min apart from Pep0, which was purified with a gradient of 5-95% over 20 min and their MS ions are summarised in Table 2.

Table 2 – MS characterisation of synthesised peptides

Peptide	Calculated mass	Calculated *m/z*	Found *m/z*	Species	HPLC t_r /min
**Pep0**	2004.4	669.1	668.6	$[M+2H]^+$	8.17
**Pep1**	1724.0	575.7	575.7	$[M+2H]^+$	6.73
**Pep2**	1752.1	585.0	584.8	$[M+2H]^+$	7.09

All constrained peptides were purified by HPLC with a gradient of 20-60% over 20 min and their MS ions are summarised in Table 3.

Table 3 – MS characterisation of synthesised constrained peptides

Constrained Peptide	Calculated Mass	Calculated *m/z*	Found *m/z*	Species	HPLC t_r/min
**Pep1A**	1859.1	620.7	620.9	$[M+3H]^+$	6.36
**Pep1B**	1887.2	630.1	630.3	$[M+3H]^+$	6.48
**Pep1C**	1915.2	639.4	939.5	$[M+3H]^+$	6.68
**Pep2A**	1887.2	630.1	630.1	$[M+3H]^+$	6.12

Peptide stapling yields are summarised in Table 4.

Table 4 – Purified yields for peptide stapling

Constrained Peptide	Percentage yield
**Pep1A**	70
**Pep1B**	73
**Pep1C**	68
**Pep2A**	57

Analytical Peptide Traces

Unconstrained Peptides Mass Spectrometry Traces

Pep0

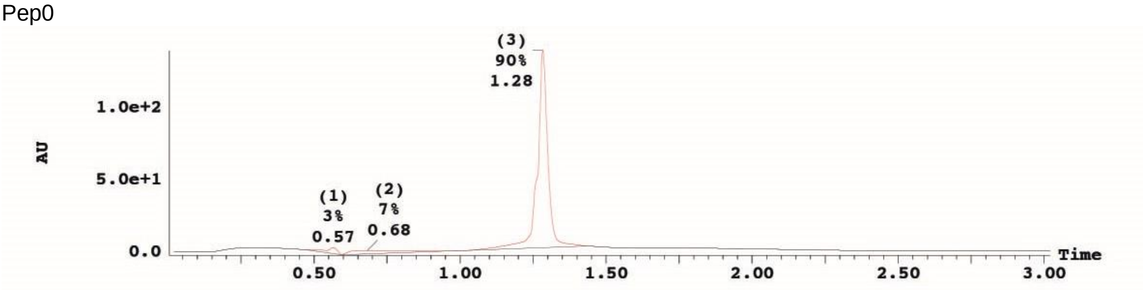

Pep1

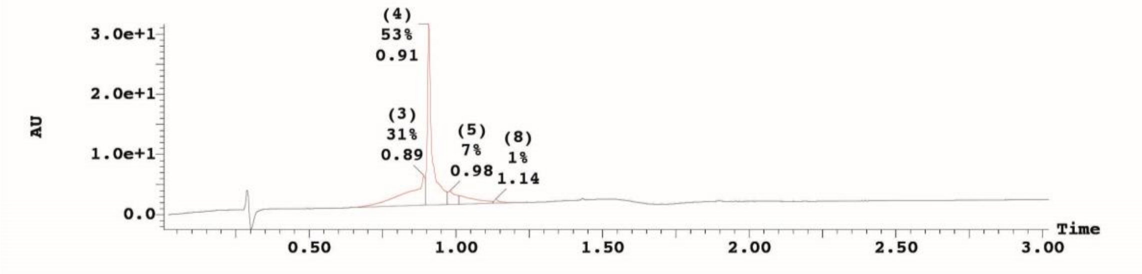

Pep2

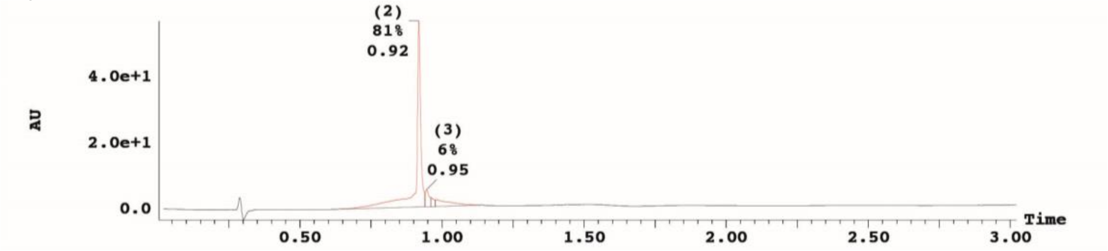

Constrained Peptides Mass Spectrometry Traces

Pep1A

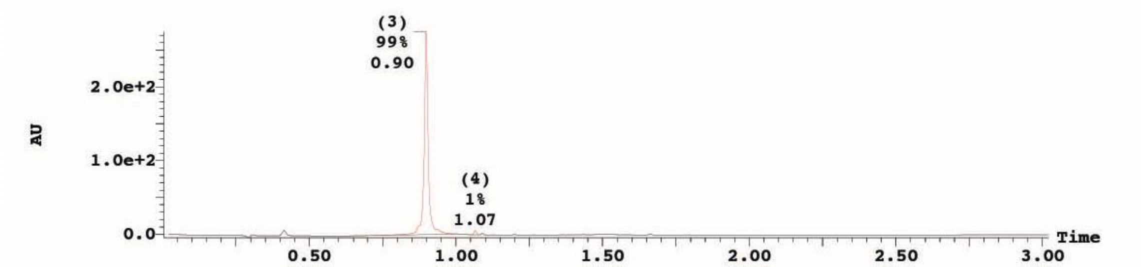

Pep1B

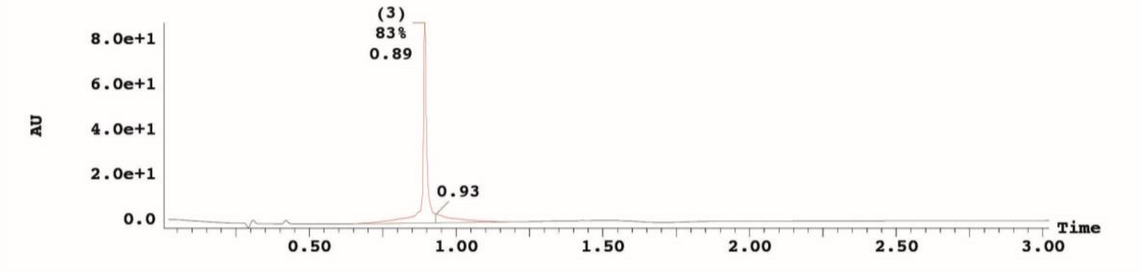

Pep1C

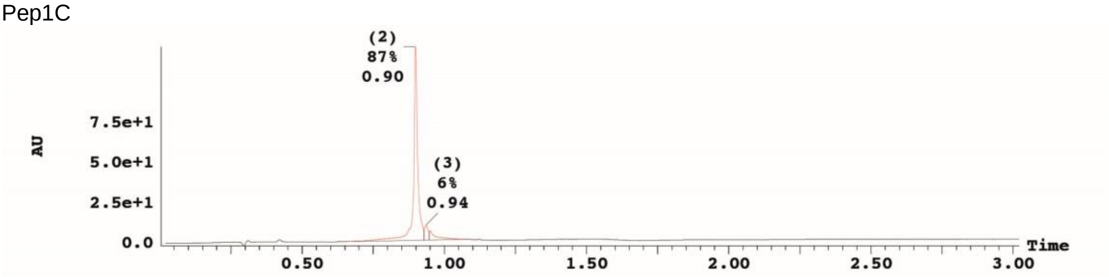

Pep2A

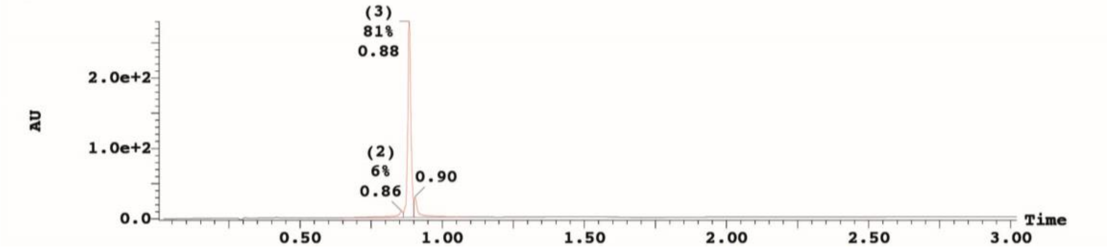

References

[1] A. Cribbs, A. Kennedy, B. Gregory, F. Brennan, *BMC Biotechnol.* **2013**, *13*, 98.
[2] R. H. Kutner, X. Y. Zhang, J. Reiser, *Nat. Protoc.* **2009**, *4*, 495-505.
[3] M. Geraerts, S. Willems, V. Baekelandt, Z. Debyser, R. Gijsbers, *BMC Biotechnol.* **2006**, *6*, 34.
[4] M. M. Wiedmann, S. Aibara, D. R. Spring, M. Stewart, J. D. Brenton, *J. Struct. Biol.*
[5] T. J. Dolinsky, J. E. Nielsen, J. A. McCammon, N. A. Baker, *Nucleic Acids Res.* **2004**, *32*, W665-W667.
[6] D. A. Case, V. Babin, J. T. Berryman, R. M. Betz, Q. Cai, D. S. Cerutti, T. E. Cheatham, III, T. A. Darden, R. E. Duke, H. Gohlke, A. W. Goetz, S. Gusarov, N. Homeyer, P. Janowski, J. Kaus, I. Kolossváry, A. Kovalenko, T. S. Lee, S. LeGrand, T. Luchko, R. Luo, B. Madej, K. M. Merz, F. Paesani, D. R. Roe, A. Roitberg, C. Sagui, R. Salomon-Ferrer, G. Seabra, C. L. Simmerling, W. Smith, J. Swails, R. C. Walker, J. Wang, R. M. Wolf, X. Wu, P. A. Kollman, University of California, San Francisco, **2014**.
[7] W. L. Jorgensen, J. Chandrasekhar, J. D. Madura, R. W. Impey, M. L. Klein, *J. Chem. Phys.* **1983**, *79*, 926-935.
[8] J. A. Maier, C. Martinez, K. Kasavajhala, L. Wickstrom, K. E. Hauser, C. Simmerling, *J. Chem. Theory Comput.* **2015**, *11*, 3696-3713.
[9] J. P. Ryckaert, G. Ciccotti, H. J. C. Berendsen, *J. Comput. Phys.* **1977**, *23*, 327-341.
[10] T. Darden, D. York, L. Pedersen, *J. Chem. Phys.* **1993**, *98*, 10089-10092.
[11] J. A. Izaguirre, D. P. Catarello, J. M. Wozniak, R. D. Skeel, *J. Chem. Phys.* **2001**, *114*, 2090-2098.
[12] H. J. C. Berendsen, J. P. M. Postma, W. F. Vangunsteren, A. Dinola, J. R. Haak, *J. Chem. Phys.* **1984**, *81*, 3684-3690.
[13] H. Gohlke, C. Kiel, D. A. Case, *J. Mol. Biol.* **2003**, *330*, 891-913.
[14] J. Srinivasan, T. E. Cheatham, P. Cieplak, P. A. Kollman, D. A. Case, *J. Am. Chem. Soc.* **1998**, *120*, 9401-9409.
[15] A. Onufriev, D. Bashford, D. A. Case, *Proteins: Struct. Funct. Bioinform.* **2004**, *55*, 383-394.
[16] M. Rarey, B. Kramer, T. Lengauer, G. Klebe, *J. Mol. Biol.* **1996**, *261*, 470-489.
[17] R. Luo, L. David, M. K. Gilson, *J. Comput. Chem.* **2002**, *23*, 1244-1253.
[18] J. Weiser, P. S. Shenkin, W. C. Still, *J. Comput. Chem.* **1999**, *20*, 217-230.
[19] W. C. Still, A. Tempczyk, R. C. Hawley, T. Hendrickson, *J. Am. Chem. Soc.* **1990**, *112*, 6127-6129.
[20] I. Massova, P. A. Kollman, *J. Am. Chem. Soc.* **1999**, *121*, 8133-8143.
[21] Y. H. Lau, D. Spring, *Synlett* **2011**, *2011*, 1917-1919.
[22] N. Fischer, E. D. Goddard-Borger, R. Greiner, T. M. Klapötke, B. W. Skelton, J. Stierstorfer, *J. Org. Chem.* **2012**, *77*, 1760-1764.
[23] R. Vinayak, *Tetrahedron Lett.* **1999**, *40*, 7611-7613.
[24] a) L. Edman, U. Mets, R. Rigler, *Proc. Natl. Acad. Sci. U. S. A.* **1996**, *93*, 6710-6715; b) G. Vámosi, C. Gohlke, R. M. Clegg, *Biophys. J.* **1996**, *71*, 972-994; c) L. Wang, A. K. Gaigalas, J. Blasic, M. J. Holden, *Spectrochim. Acta, Pt. A: Mol. Biomol. Spectrosc.* **2004**, *60*, 2741-2750.
[25] A. Brunner, Y. Minamitake, A. Göpferich, *Eur. J. Pharm. Biopharm.* **1998**, *45*, 265-273.
[26] a) C. Larkin, S. Datta, M. J. Harley, B. J. Anderson, A. Ebie, V. Hargreaves, J. F. Schildbach, *Structure* **2005**, *13*, 1533-1544; b) J. R. Unruh, G. Gokulrangan, G. H. Lushington, C. K. Johnson, G. S. Wilson, *Biophys. J.* **2005**, *88*, 3455-3465; c) B. J. Anderson, C. Larkin, K. Guja, J. F. Schildbach, in *Methods Enzymol., Vol. Volume 450*, Academic Press, **2008**, pp. 253-272.
[27] M. V. Kvach, I. A. Stepanova, I. A. Prokhorenko, A. P. Stupak, D. A. Bolibrukh, V. A. Korshun, V. V. Shmanai, *Bioconj. Chem.* **2009**, *20*, 1673-1682.
[28] J. E. T. Corrie, J. S. Craik, *J. Chem. Soc., Perkin Trans. 1* **1994**, 2967-2973.
[29] M. Amblard, J.-A. Fehrentz, J. Martinez, G. Subra, *Mol. Biotechnol.* **2006**, *33*, 239-254.
[30] T. Christensen, *Acta Chem. Scand.* **1979**, *33b*, 763-766.
[31] A. J. Kimple, A. Yasgar, M. Hughes, A. Jadhav, F. S. Willard, R. E. Muller, C. P. Austin, J. Inglese, G. C. Ibeanu, D. P. Siderovski, A. Simeonov, *Combinatorial Chem. High Throughput Screening* **2008**, *11*, 396-409.
[32] a) J. Qi, M. Oppenheimer, P. Sobrado, *Enzyme Research* **2011**, *2011*, 9; b) W. A. Lea, A. Simeonov, *Exp. Op. Drug Disc.* **2011**, *6*, 17-32.
[33] a) B. Kobe, *Nat. Struct. Mol. Biol.* **1999**, *6*, 388-397; b) P. Fanara, M. R. Hodel, A. H. Corbett, A. E. Hodel, *J. Biol. Chem.* **2000**, *275*, 21218-21223; c) J. K. Forwood, V. Harley, D. A. Jans, *J. Biol. Chem.* **2001**, *276*, 46575-46582.
[34] S. I. van Kasteren, H. B. Kramer, H. H. Jensen, S. J. Campbell, J. Kirkpatrick, N. J. Oldham, D. C. Anthony, B. G. Davis, *Nature* **2007**, *446*, 1105-1109.
[35] F. Giordanetto, J. D. Revell, L. Knerr, M. Hostettler, A. Paunovic, C. Priest, A. Janefeldt, A. Gill, *ACS Med. Chem. Lett.* **2013**, *4*, 1163-1168.
[36] 菊. 佐藤, *日本化學雜誌* **1956**, *77*, 1411-1413.
[37] R. P. Verma, A. Shandilya, V. Haridas, *Tetrahedron* **2015**, *71*, 8758-8765.
[38] R. Gelin, J. C. Hablot, *Bull. Soc. Chim. Fr.* **1966**, 3079-3082.
[39] J. C. Marine, G. Lozano, *Cell Death Differ.* **2010**, *17*, 93-102.
